# Visual exposure to buildings in Switzerland: spatial patterns and changes over six decades

**DOI:** 10.1038/s41598-025-18772-7

**Published:** 2025-10-07

**Authors:** Andreas Moser, Adrienne Grêt-Regamey

**Affiliations:** Planning of Landscape and Urban Systems (PLUS), ETH Zurich, Zurich, Switzerland

**Keywords:** Visual exposure assessment, Viewshed analysis, Cumulative visibility, Rural development, Spatial planning, Ecology, Ecology, Environmental sciences, Environmental social sciences, Geography, Geography

## Abstract

The visual presence of buildings in rural landscapes is a key yet often overlooked dimension of spatial development. This study presents a comprehensive visual exposure assessment of rural buildings, defined here as buildings located outside building zones, across Switzerland from 1960 to 2024, using binary and cumulative viewshed analyses. By integrating historical and contemporary building footprint datasets with digital height models and forest cover data, we quantify how visibility to buildings has evolved across five biogeographical regions. Results reveal a steady increase in visual exposure, primarily driven by construction in non-building zones, despite regulatory efforts to contain urban sprawl. The Plateau region exhibits the highest cumulative visibility due to its dense settlement and open terrain, while the Alpine regions maintain significant areas with minimal visual intrusion. Temporal patterns show that the most significant increases in visibility occurred between 1960 and 1980, aligning with broader trends of urban sprawl. The findings underscore the importance of incorporating visual exposure metrics into spatial planning and landscape monitoring frameworks. This study contributes a visibility-based perspective to the assessment of rural development and offers a valuable tool for evaluating the visual quality of changing landscapes.

**Supplementary Information:**

The online version contains supplementary material available at 10.1038/s41598-025-18772-7.

## Introduction

Rural landscapes across Europe are undergoing significant transformation due to urbanisation pressures, agricultural development, and evolving spatial planning policies^1^. While much attention has been paid to the ecological and functional consequences of dispersed development, its visual dimension, how buildings alter the perceptual experience of rural landscapes, has received comparatively little focus^2^. Understanding the spatial patterns, spatial configurations, and physical characteristics of landscapes is crucial for informed decision-making in areas such as environmental planning, urban development, and ecological research^3,4^. Visual landscape analysis has emerged as a key practice for quantifying, representing, and interpreting the perceived and experienced qualities of a landscape^5^. The basis for this practice is a viewshed analysis, a computational method for identifying the areas visible from a specific location based on topographical and terrain features^6^.

Viewshed analysis has been applied widely across numerous disciplines. In landscape planning and management, it is used to integrate aesthetic considerations, investigate visual exposure, and assess the distribution and quality of visual landscape properties^7–9^. This extends to evaluating the visual exposure of proposed infrastructure, such as wind turbines^10,11^ solar farms^12,13^ highways^14^ and buildings^15^ allowing for informed decision-making and, thus, the minimisation of negative consequences. Tourism and recreation planning also leverage visibility analysis to design scenic routes, optimise viewpoints, and enhance the experience of visitors^16,17^. In the real estate sector, the visibility of natural landscapes can be evaluated to reveal the influence on property values^18,19^. Beyond these domains, recent studies in environmental psychology and urban health have used viewshed-based approaches to quantify eye-level greenness visibility, showing how visual access to greenery can reduce stress and enhance well-being^20,21^. Furthermore, archaeologists employ viewsheds to explore the intervisibility between sites and gain insights into past cultural perceptions of landscapes^22^.

Alongside the diverse applications, methodological advances have expanded the scope offering a richer understanding of landscape visibility. Moving beyond the visible and non-visible dichotomy, fuzzy viewsheds account for uncertainty in the data or atmospheric conditions by assigning a probability or degree of visibility to each location^17,23^. Cumulative viewsheds are generated by aggregating the visibility from multiple viewpoints^24^ indicating the number of times a location is visible, and can be used to infer cumulative visual impacts^5^. The visual magnitude further refines visibility analysis by quantifying the space a visible area occupies within an observer’s view, considering factors such as the slope, angle, and distance^25–27^. This provides a measure of the degree to which a structure is visible and potentially attention-grabbing^14^. More recently, the concept of *viewscapes* has emerged, aiming to characterise not merely that which is visible but how humans visually connect to their surrounding environment, considering the contents of the visible area and human perception^19,28^. Because such analyses can be computationally demanding, especially when involving large datasets or many observer points, research has also advanced on the algorithmic side. Developments such as the HiXDraw algorithm^29^ and parallel computing using graphics processing units (GPUs)^30^ have substantially improved the efficiency of large-scale visibility assessments.

Despite these advances, relatively few studies have addressed the dynamic nature of visibility across spatial and temporal dimensions. Most visual landscape assessments rely on static representations, typically derived from isolated observer points at a single moment in time. Studies over longer time periods, such as the assessment of evolving visual impacts from wind farm development^10^ remain rare. While such approaches are valuable for individual impact assessments, they offer limited insight into how visibility evolves as landscapes are reshaped by land use changes. As a result, visibility metrics—although frequently applied in infrastructure and energy planning case studies—are rarely integrated into broader discourses on rural landscape transformation and settlement development, particularly in relation to spatial planning policies. This lack of spatio-temporal perspective constrains our ability to anticipate long-term or cumulative visual impacts^31^.

This study investigates how the visual exposure to rural buildings in Switzerland has evolved over the past six decades. Specifically, we apply binary and cumulative viewshed analyses to quantify the spatial and temporal dynamics of building visibility across five biogeographical regions, distinguishing between buildings located within and outside of designated building zones. By integrating historical cartographic data with modern geospatial techniques, we aim to provide a spatially explicit, visibility-based assessment of rural and settlement development. This approach not only enhances our understanding of visual landscape change but also offers valuable insights for integrating visibility metrics into spatial planning and landscape monitoring frameworks.

The paper is organised into five sections. Following this introduction, section “Materials and methods” details the data sources and methodological approach employed in the analysis. Section “Results” reports the empirical results of the spatial and temporal assessments. Section “Discussion” situates these findings within broader theoretical, planning, and policy contexts. Finally, section “Conclusion” concludes by summarising the main contributions of the study and outlining avenues for future research.

## Materials and methods

### Study area

**Fig. 1:**
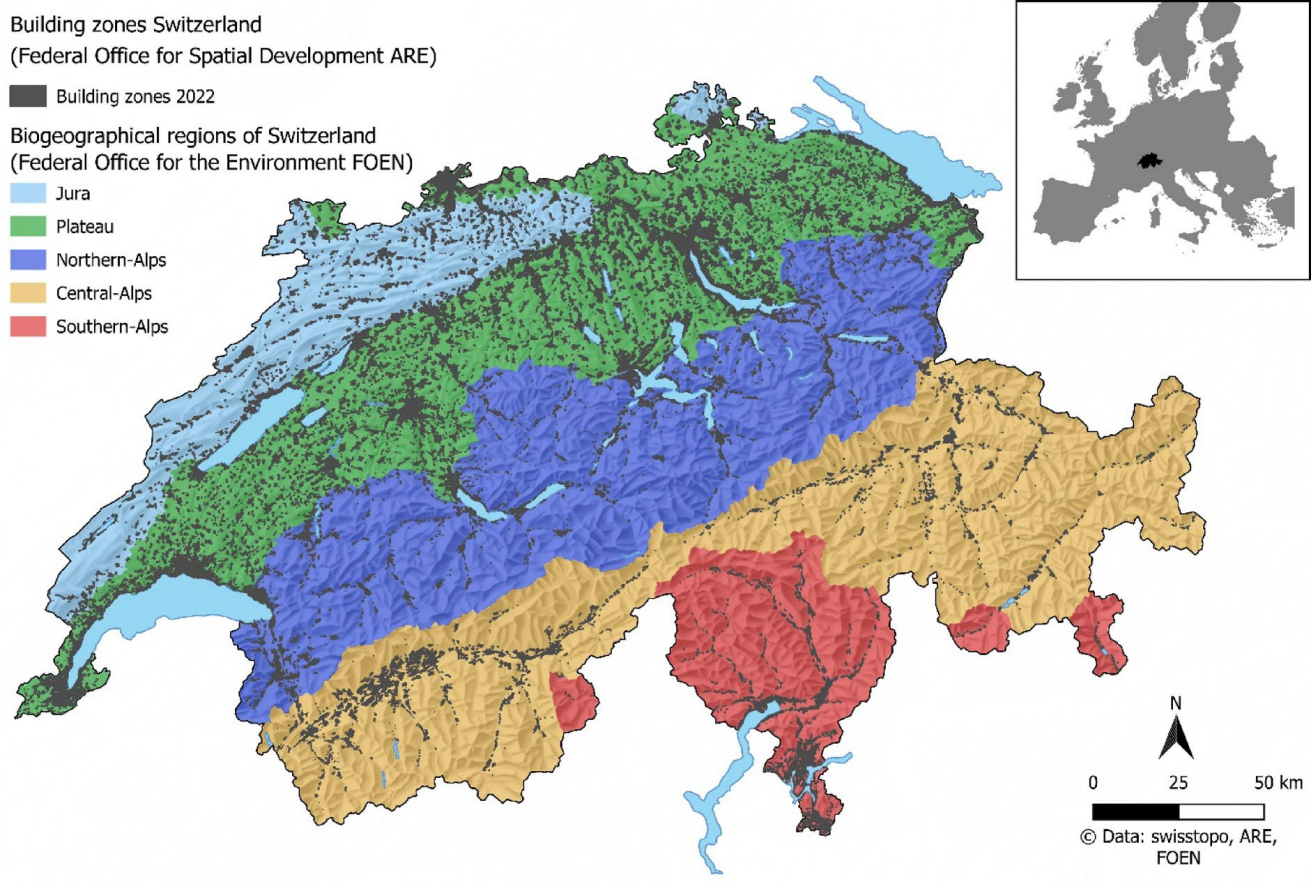
Study area of Switzerland with building zones of 2022 and biogeographical regions. Map created using QGIS v3.22.5 (https://qgis.org).

Switzerland is in central Europe and has a population of approximately 8.8 million and a national average population density of 212 people per square kilometre (see Fig. 1). Over the past ten years, the country has seen a 9.7% demographic growth^32^. By contrast, the European Union has an average population density of just 109 inhabitants per square kilometre, and experienced a more modest population increase of 1.3% during the same period^33,34^. A comparative analysis by Hennig et al.^35^ places Switzerland among the top third of European countries in terms of *urban sprawl*, defined as low-density, spatially dispersed development. This phenomenon is widespread across Europe and is particularly evident near metropolitan areas, along major transport routes, and in regions with high levels of industrialisation.

Like many other countries in Europe, Switzerland has implemented urban growth boundaries (i.e., building zones to contain urban sprawl) and promotes the densification of already built areas^36^. The Swiss Spatial Planning Law, enacted in 1980, designates building zones to concentrate urban development; create compact settlements; and protect natural resources, such as soil, air, water, forests, and landscapes^37^. In contrast, areas outside these zones are classified as non-building zones, where development is subject to strict limitations. In this study, the term *rural areas* refers to these non-building zones, which cover 95% of the country’s territory and hold 5% of the population^38^. Construction outside of building zones is restricted by the Swiss Spatial Planning Act, only allowing for the renovation or construction of agricultural buildings. In addition, certain exemptions are made for existing buildings and new site-specific constructions and infrastructure, such as roads, quarries, and buildings for energy production. Despite the restrictions and a recent trend towards densification, the settlement area outside the building zones has experienced continuous growth, reaching 36% of Switzerland’s total settlement area today^39^.

### Data sources

The basis for the visual exposure assessment of buildings over time involves vector data of building footprints. Data originate from segmented and vectorised analogue maps for the time steps of 1960, 1970, 1980, and 1990, as well as from digital cartographic products for the time steps of 2004, 2014, and 2024. In the following section, the derivation of building footprint datasets is detailed for the early historical and digital time steps, outlining the sources and processing that provided the highest comparability in terms of completeness and level of building detail across time.

For the early time steps (i.e., 1960, 1970, 1980, and 1990), building footprints were derived from historic National Map sheets at the scale of 1:25’000 that have been scanned into raster format by the Swiss Federal Office of Topography swisstopo^40^. Raster data with segmented buildings from the National Map were obtained from the study by Räth et al.^41^ who adapted a U-Net segmentation algorithm originally developed by Heitzler and Hurni^42^. The segmented buildings were vectorised, and the resulting building footprints were regularised using the corresponding ArcGIS Pro tool to remove undesirable rasterisation artefacts in the geometries^43^.

The first complete edition of the 249 individual map sheets (sheet format of 70 × 48 cm corresponding to 3’360 km^2^ of the National Map covering the entire country was produced between 1952 and 1979. Existing map sheets were already updated during this phase, and upon the publication of the last map sheet of the first edition, a regular six-year updating cycle, which is still valid today, was launched. Due to this production cycle, it was not possible to generate nationwide building footprint data with the exact reference year. Thus, the time steps of 1960, 1970, 1980, and 1990 represent an assembly of map sheets with varying years of issue surrounding the referenced time step (cf. Supplementary material Figure S1). To avoid using the same map sheet for multiple time steps, a selection strategy was applied that prioritised temporal separation between the chosen versions for each map sheet. Nonetheless, for six map sheets, only three distinct years of issue were available, requiring the reuse of the same map sheet for two different time steps (cf. Supplementary material Figure S2).

The U-Net model has been shown to perform well in segmenting cartographic maps, with applications to Swiss Siegfried maps achieving an average intersection over union of 88.2% and a precision of 98.55%^42^. Nevertheless, the authors noted that the highest risk of false building detections arises from rock hachures, i.e., striped and shaded patterns used to represent rocky terrain in mountainous areas. To address this issue, a plausibility check involving manual verification was conducted, as falsely recognised buildings in remote, otherwise unsettled areas could significantly impact the viewshed analysis (see section “Temporal and spatial visibility analysis of buildings”). For each building, the minimal distance to any building in the subsequent time step was calculated. Buildings with a minimum distance greater than 500 m were manually compared against the original National Maps or historical aerial photographs to assess their validity and were removed from the dataset if confirmed to be false classifications. Across the 1960–1990 time steps, this procedure resulted in the removal of 0.12% of the initially classified buildings.

At the turn of the century, digital cartographic products started to replace analogue maps, and in 1995, swisstopo started to digitalise the National Maps at a scale of 1:25’000^44^. In the resulting VECTOR25 dataset, nationwide vectorised building footprints first became available in 2004^45^. As a direct derivation of the analogue National Map, VECTOR25 offers a level of detail in building geometries comparable to segmented and vectorised building data. These data were used without any processing for the time step of 2004.

With ongoing digitalisation efforts, the VECTOR25 dataset was discontinued and transferred to the new large-scale swissTLM3D topographic landscape model, featuring a higher level of detail with minimal cartographic generalisation of building outlines^46^. In the new swissTLM3D product, building roofs are projected onto the underlying terrain model to generate polygonal building footprints^47^. This results in overlapping polygons and significantly more objects than in the VECTOR25 data. In preparation for the time step of 2014, all intersecting building footprints of the swissTLM3D model were, therefore, merged.

Since 2016, swisstopo has provided a vectorial form of the National Map (1:25’000), known as Swiss Map Vector 25^48^. In this product, building footprints are represented with high positional accuracy but in a more generalised form compared to the more detailed swissTLM3D dataset. Buildings from the Swiss Map Vector 25 data were selected for the time step of 2024.

To support a differentiated understanding of rural development, all buildings were classified based on whether they are located within the most recently available building zones as of 2022 or in non-building zones^38^. For further spatial analysis, the buildings were allocated to the biogeographical regions of Switzerland^49^. The Western Central-Alps and Eastern Central-Alps were merged to arrive at the five areas of Jura, Plateau, Northern-Alps, Central-Alps, and Southern-Alps (see Fig. 1).

### Temporal and spatial visibility analysis of buildings

**Fig. 2:**
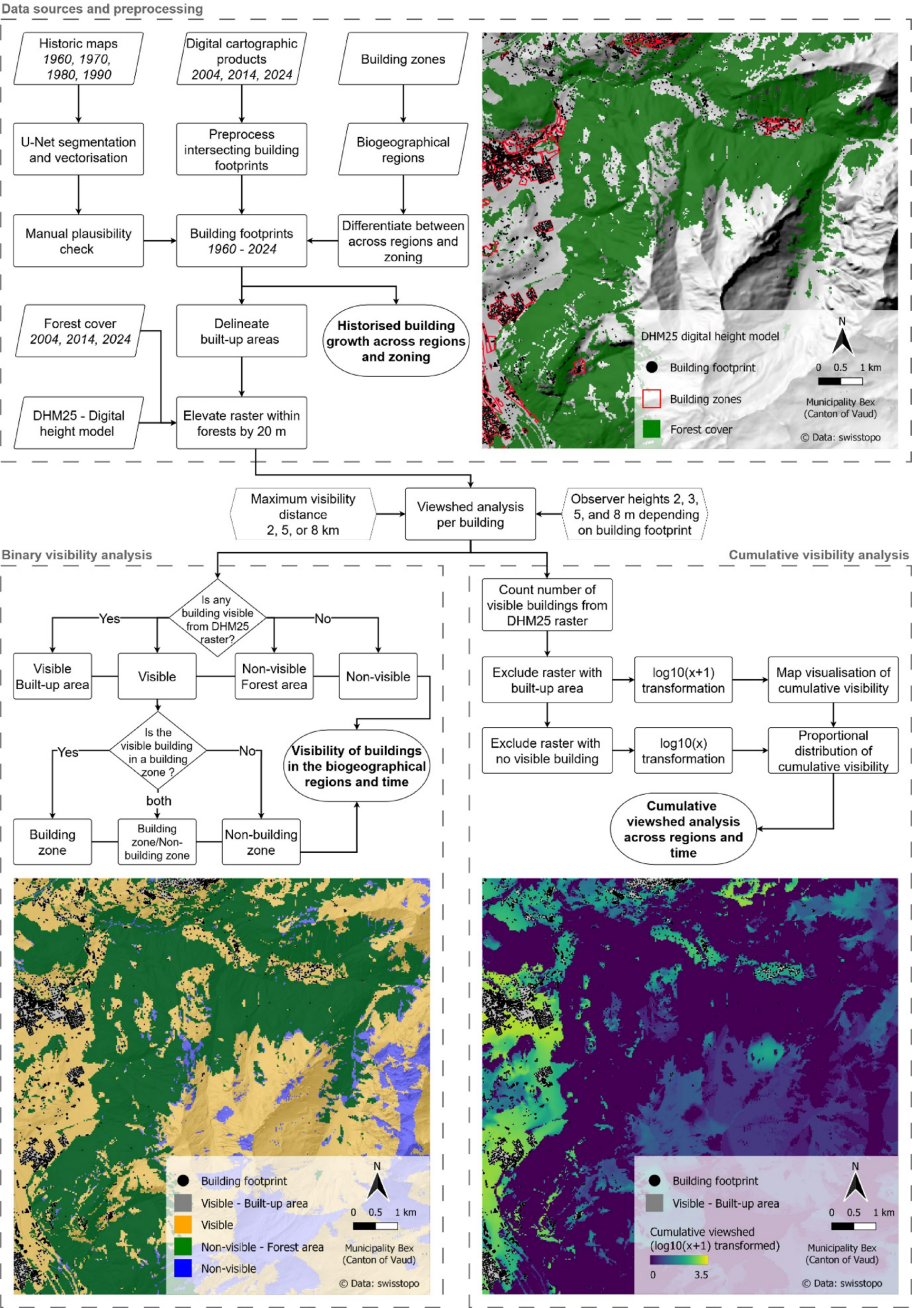
Overview of the methodological workflow including the preprocessing of datasets, binary, and cumulative visibility analysis. Maps created using QGIS v3.22.5 (https://qgis.org).

The viewshed analysis was conducted using the raster-based digital height model DHM25, with a resolution of 25 m, provided by the Swiss Federal Office of Topography swisstopo^50^. The visibility of each building was computed in the unbuilt open landscape up to a maximum distance of 2 km (in addition to 5 km and 8 km to assess sensitivity to this parameter). Initially, the outcome was transposed into a binary analysis^6^ indicating whether buildings were visible for every raster cell or not. Rather than modelling the views accessible from individual observer points or from buildings, our analysis focuses on the extent to which buildings themselves are visually prominent within the surrounding open landscape. This analysis further distinguished whether visible buildings are located within or outside of the 2022 building zones^38^. Complementing this approach, a cumulative visibility analysis was performed to determine the number of visible buildings from each raster cell^14,24,51^. An overview of the methodological workflow, including the preprocessing of datasets, as well as the distinction between binary and cumulative visibility analyses, and the applied parameters, is provided in Fig. 2.

The binary and cumulative visibility were calculated across the entire landscape; however, meaningful interpretations were limited to open, unbuilt areas where the line of sight is not obstructed by settlements or forests. Built-up areas were delineated for each time step using the cartographic functions in ArcGIS Pro^43^. In the binary analysis, built-up areas are simply visible areas. In the cumulative visibility analysis, however, the number of buildings identified as visible within these built-up areas is not realistic because the buildings themselves obstruct views, an effect not captured by the digital height model. Therefore, built-up areas were excluded from the cumulative analysis to ensure a more realistic result, with emphasis on visible open, unbuilt areas.

Like built-up areas, forests are not considered by the DHM25 digital height model, which represents the Earth’s surface without buildings or vegetation. However, forests can block lines of sight, particularly in flatter regions, where building visibility would be overestimated^52^. To address this limitation, all raster cells located within forest areas were elevated by 20 m in the model and considered to have no line of sight to any buildings^53^. Forest cover data are available for the years 2004, 2014, and 2024 through the VECTOR25 and swissTLM3D datasets^45,47,54^. For the earlier time steps (1960, 1970, 1980, and 1990), the forest cover of 2004 was used to modify the digital height model accordingly. The effect of an increasing forest area over time was only considered in the binary viewshed analysis; in contrast, the cumulative viewshed analysis was conducted using a constant forest cover from 2024. This approach allowed for the assessment of building development over time without the confounding influence of a changing forest cover. While this assumption simplifies the interaction between vegetation growth and visibility, additional analyses for 2004, 2014, and 2024 using changing forest cover are provided to illustrate its effects.

The maximum visibility distance (2 km) in this study was selected based on a combination of atmospheric, perceptual, and contextual considerations. Atmospheric conditions such as relative humidity and particulate matter restrict actual visibility far more than the physiological limits of human vision^15^. According to a report from Grange and Hüglin^55^ the average horizontal visibility across Switzerland varies significantly depending on the season and locality, ranging between approximately 10 and 40 km. However, the visual significance of an object decreases with distance due to the factors of relative size and contrast^12,27^. To account for this effect, landscape assessment studies often apply distance decay functions^13,17,25^ or consider shorter so-called *middle-distances*, as defined by Higuchi^56^. According to Higuchi^56^ the middle-distance view is what people typically associate with a pictorial landscape, where “variations in the shape of the terrain become important compositional elements [and] we see the forest rather than the trees”. Finally, while not directly related to conventional visibility analysis, the scale of perception is also reflected in urban sprawl metrics. In this context, Jaeger and Schwick^57^ considered a *horizon of perception* of 2 km to be particularly suitable for Switzerland, as this corresponds to the spatial scale at which people can perceive urban sprawl.

As historical maps and earlier vector datasets do not provide information on building heights, these have been consistently approximated across all time steps as a function of the building footprint size. Buildings with footprint areas less than 100, 500, and 1’000 m^2^ were assigned observer heights of 2, 3, and 5 m, respectively, while buildings with footprint areas greater than 1’000 m^2^ were assigned a height of 8 m. These values were chosen as simplified but realistic approximations of common building types in Switzerland, ranging from small sheds or garages (2–3 m) to single-story (5 m) and two-story buildings (8 m). Although this approach does not account for the trend towards taller buildings and skyscrapers, such buildings are typically located in urban centres and are unlikely to significantly affect visibility outcomes in open, unbuilt rural landscapes. Given the defined maximum visibility distance, any potential influence of these taller buildings would be limited to the peripheries of built-up areas.

The computationally demanding viewshed tasks were performed with GPU-enhanced geospatial analysis using the xarray-spatial Python package^58^.

## Results

### Historised Building growth across regions and zoning in Switzerland

**Fig. 3:**
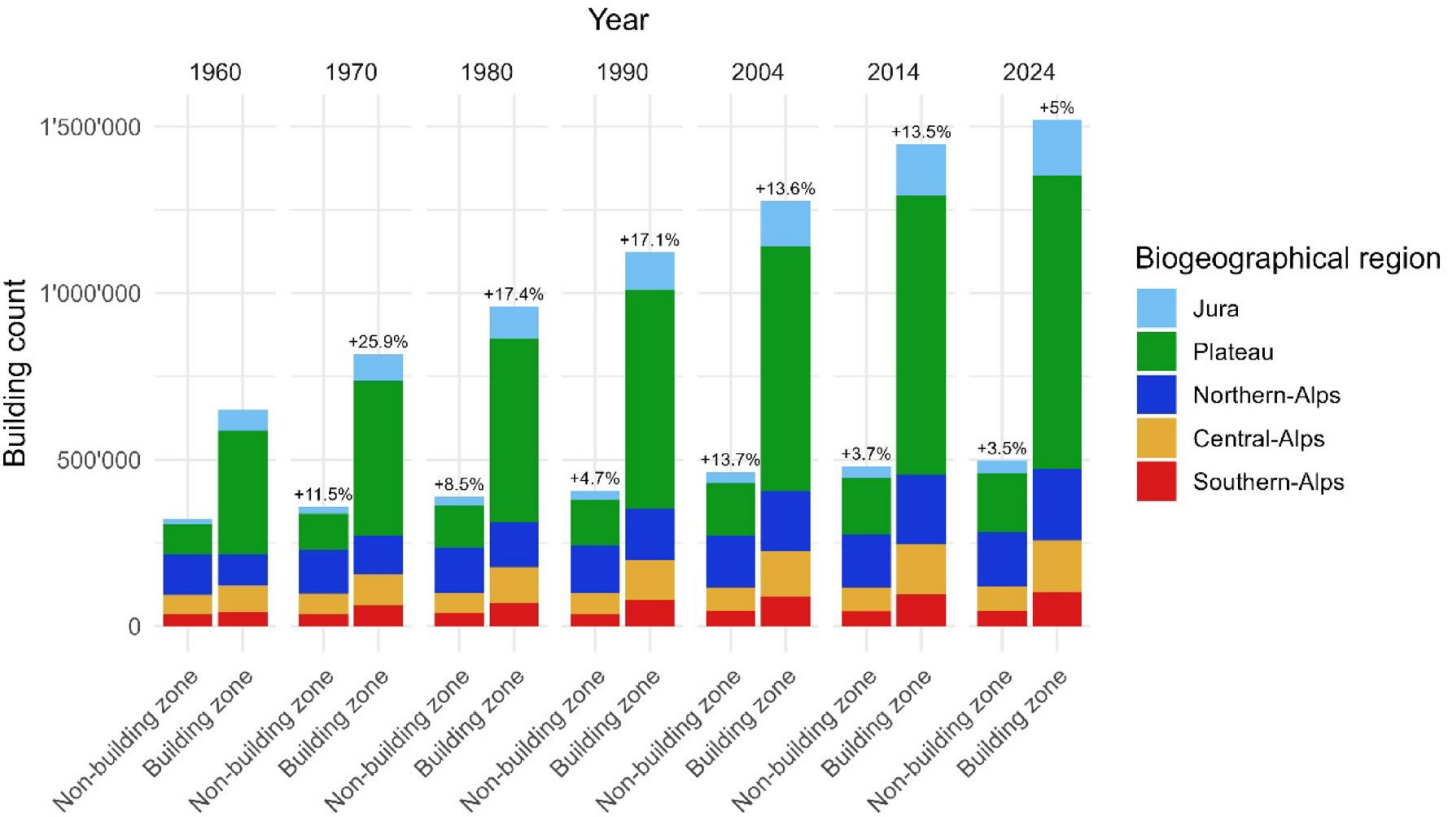
Temporal and spatial increases in building numbers of the biogeographical regions of Switzerland, categorised according to building zones and non-building zones issued in 2022.

The number of buildings in Switzerland increased steadily from 1960 to 2024, with substantial regional variations (Fig. 3; see also Supplementary material Table S1 for detailed data according to biogeographical regions, building zones and non-building zones). Overall, the building number more than doubled, with 971’869 in 1960 and 2’027’426 in 2024. The Plateau region consistently exhibited the highest proportion of buildings, accounting for over 47% of the total throughout the period and reaching 52.4% by 2024. The Jura and Southern-Alps showed lower proportions, ranging between 8% and 10%. The Northern-Alps maintained a relatively stable share, slightly declining from 22.3% in 1960 to 18.7% in 2024, whereas the Central-Alps saw a gradual decrease from 14 to 11.3% during the same timeframe. These trends indicate a high concentration of building activities within the Plateau, accompanied by continuous but slower growth in the Alpine regions, resulting in declining relative shares.

Between 1960 and 2024, the growth rate of buildings generally decreased across all regions; the Jura region showed a significant initial growth of 32.2% between 1960 and 1970, declining steadily thereafter to 9.2% in the most recent decade (2014–2024). The Plateau region displayed a similar pattern, with a pronounced early growth of 23.8% (1960–1970), gradually decreasing and reaching a mere 4.8% between 2014 and 2024. The Alpine regions followed comparable trends, although with more variability. The Northern-Alps experienced a consistent reduction in growth rates, from an initial 14.8% (1960–1970) down to a mere 2.6% (2014–2024). Similarly, the Central-Alps and Southern-Alps exhibited high initial growth rates of 13.6% and 24.8%, respectively (1960–1970), with subsequent reductions to 3.5% and 4.5%, respectively, in the most recent period.

This general pattern of a declining growth rate was also observed when differentiating between the most recent building zones and non-building zones of 2022. Growth rates within building zones consistently surpassed those in non-building zones (cf. Fig. 3). For instance, the growth rate in building zones across Switzerland was initially high (25.9%) between 1960 and 1970, decreasing steadily to 5% between 2014 and 2024. In contrast, the corresponding growth rates for non-building zones were consistently lower, starting at 11.5% from 1960 to 1970 and declining to 3.5% in the most recent decade. In some cases, non-building zones even exhibited negative growth, particularly evident in the Southern-Alps from 2004 to 2014 (− 1.3%), coinciding with a change in the underlying data sources. Despite lower growth rates, the absolute number of buildings in non-building zones increased steadily over the entire period.

At the national level, the share of buildings located within the 2022 building zones increased slightly from 66.8% in 1960 to 75.3% in 2024, while the share in non-building zones decreased from 33.2 to 24.7%, based on the mentioned data sources.

### Visibility of buildings in the biogeographical regions of Switzerland

**Fig. 4:**
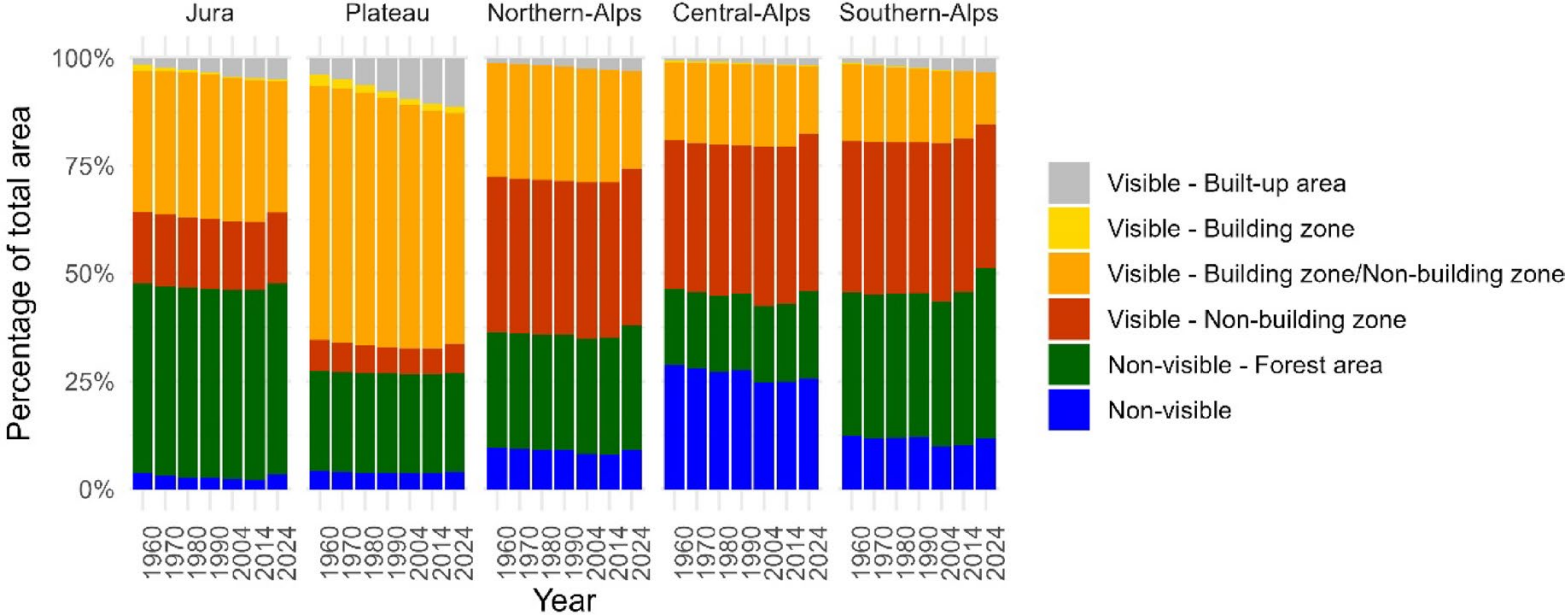
Percentage of biogeographical regions of Switzerland visible or non-visible to buildings.

Distinct spatial patterns in the visibility of buildings in landscapes are apparent across Switzerland’s biogeographical regions, and these patterns remain largely consistent over time (Fig. 4). Detailed proportions and the corresponding areas of each biogeographical region are provided in Supplementary material Table S2.

The Plateau region exhibits the highest proportion of visible areas. Using 2024 as a reference point, visible built-up areas account for 11.3%, and an additional 61.7% of unbuilt landscape is visible to buildings, mostly involving buildings in building zones and non-building zones. The remaining non-visible area is predominantly forested, covering 23.1% of the region, while only 3.9% constitutes open, non-forested landscapes with no visibility to buildings.

In contrast to the Plateau, the Alpine regions, including the Northern, Central, and Southern-Alps, are characterised by substantially lower visibility to buildings, reflecting their topographical complexity and more limited built-up settlement areas. In 2024, visible built-up areas in the Central-Alps accounted for only 1.7% of the total area, with an additional 52.2% of the landscape visible to buildings, primarily from non-building zones (36.6%) and areas visible to buildings across both zones (15.6%). The Northern- and Southern-Alps show similar proportions, with slightly more visible built-up areas at 3.0% and 3.2%, respectively. The total visible areas reached 58.9% in the Northern-Alps and 45.2% in the Southern-Alps, mostly due to buildings located in non-building zones (36.1% and 33.2%, respectively). Non-visible areas remain significant across these regions, with the Central-Alps retaining a combined 45.8% of forested (20.1%) and open non-visible (25.7%) areas. The Northern-Alps exhibit a non-visible area of 38.1%, with 29.1% comprising forests and 9.0% constituting open landscapes, while the Southern-Alps retain the highest proportion of non-visible landscapes at 51.4%, mostly due to forests (39.6%) and open terrain (11.8%).

The Jura shows an intermediate visibility pattern compared to the Plateau and Alpine regions. Built-up areas reached 5% in 2024, with landscapes visible to buildings from building zones and non-building zones accounting for 30.3% of the region; this is higher than the value in the Alps but notably lower than the value for the Plateau. Visibility from non-building zones alone stands at 16.5%, which is less than half the value observed in the Alpine regions. Non-visible areas (47.2%) are prevalent in the Jura, but most constitute forests, and only 3.4% comprise non-forested landscapes.

**Fig. 5:**
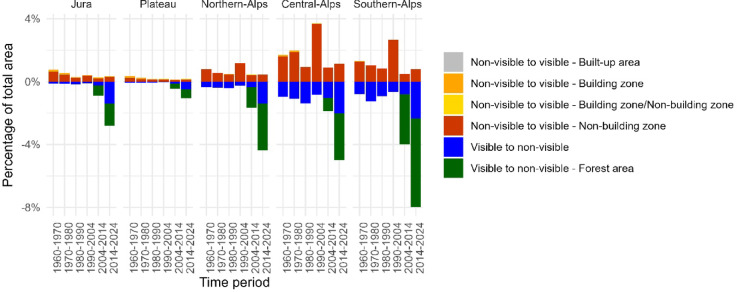
Transitions between visible and non-visible categories over different time periods.

The basic distinction between visible and non-visible areas remained relatively stable throughout the observation period. Most apparent in Fig. 4 is the expansion of visible built-up areas, often extending into the immediate surroundings of other already visible areas. Additionally, an increase in forest cover is evident from 2004 onwards, marking the earliest availability of forest data (see section “Temporal and spatial visibility analysis of buildings”). To illustrate the dynamics in more detail, Fig. 5 depicts the net transitions between visible and non-visible areas, excluding transitions where the visibility remains unchanged (e.g., visible unbuilt areas becoming built-up or non-visible open land becoming forested). These opposite transitions reveal offsetting patterns in which visibility increases and decreases at different locations partially balance each other out.

In all regions, there is a small but persistent trend of non-visible areas becoming visible, almost exclusively due to buildings in non-building zones (Fig. 5; see also Supplementary material Table S3 for more detail). In the Jura and Plateau, where the proportion of non-visible areas is already very small, this transition lies in the range of 0.2–0.6% and 0.1–0.2%, respectively. In the Alpine regions, which include larger areas of non-visible land, these transitions are higher. The Northern, Central, and Southern-Alps regularly show increases in visibility to buildings in non-building zones in the range of 0.4–1.3% per period. While higher values appeared between 1990 and 2004, especially in the Central and Southern-Alps, these were likely influenced by the change in building footprint data sources from the segmented analogue National Maps to vector data during that period; thus, they should be interpreted with caution (see Section Data sources2.2).

Conversely, the transitions from visible to non-visible areas have become more noticeable since 2004 and are closely tied to forest growth. Between 2014 and 2024, the total percentage of visibility transitions to non-visible areas reached 2.8% in the Jura, 4.4% in the Northern-Alps, 5.0% in the Central-Alps, and 7.9% in the Southern-Alps. In many cases, these transitions align almost entirely with forest expansion, such as in the Southern-Alps, where 5.6% of the landscape became forest cover and the remaining 2.3% was mainly obscured by forests in this period alone. Prior to 2004, when no distinct forest data were available, the level of visibility transitions to non-visible areas across all regions was small, typically ranging from 0.1 to 1.4% per time period, with the highest values identified in the Central-Alps. Besides the possibility of falsely segmented buildings in the data during the period from 1960 to 1990, these small transitions arose due to abandoned ruined alpine huts not mapped anymore. A unique case of visibility transitions involves the flooding of valleys due to dam construction and the submersion of an entire alp (Lai da Sontga Maria, completed in 1968).

Finally, increasing the parameter of the maximum visibility distance of buildings from 2 km to 5 km or 8 km changes the proportions of visible and non-visible areas (cf. Supplementary material Figure S3 to Figure S6). However, this does not significantly impact the overall patterns. Temporal trends, regional differences, and the relative contributions of buildings within and outside of building zones remain consistent across these parameter settings.

### Cumulative viewshed analysis across regions and time

**Fig. 6:**
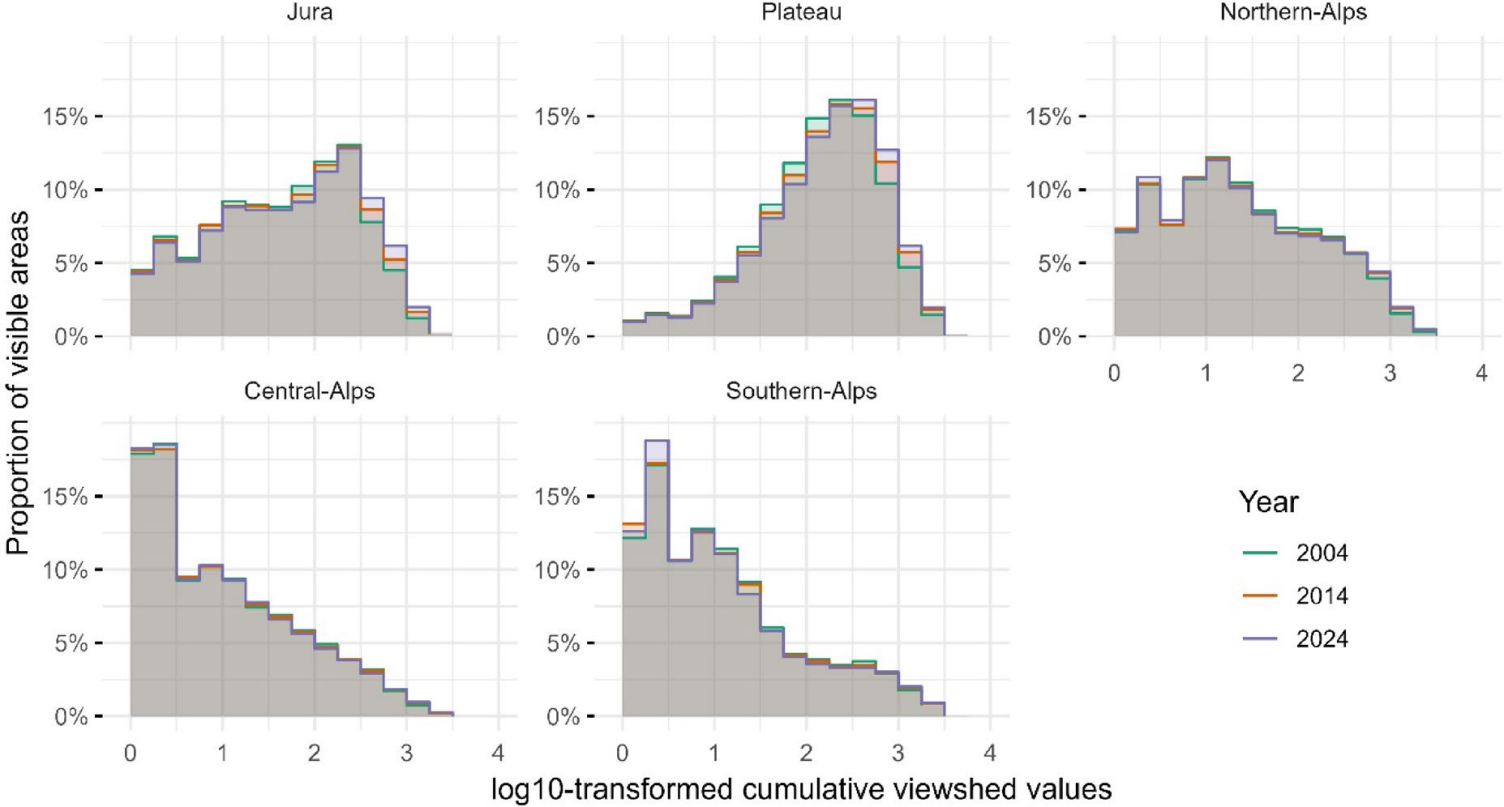
Percentage distribution of visible areas across log10-transformed bins of cumulative building visibility in the biogeographical regions of Switzerland for the years 1960, 1970, 1980, and 1990.

**Fig. 7:**
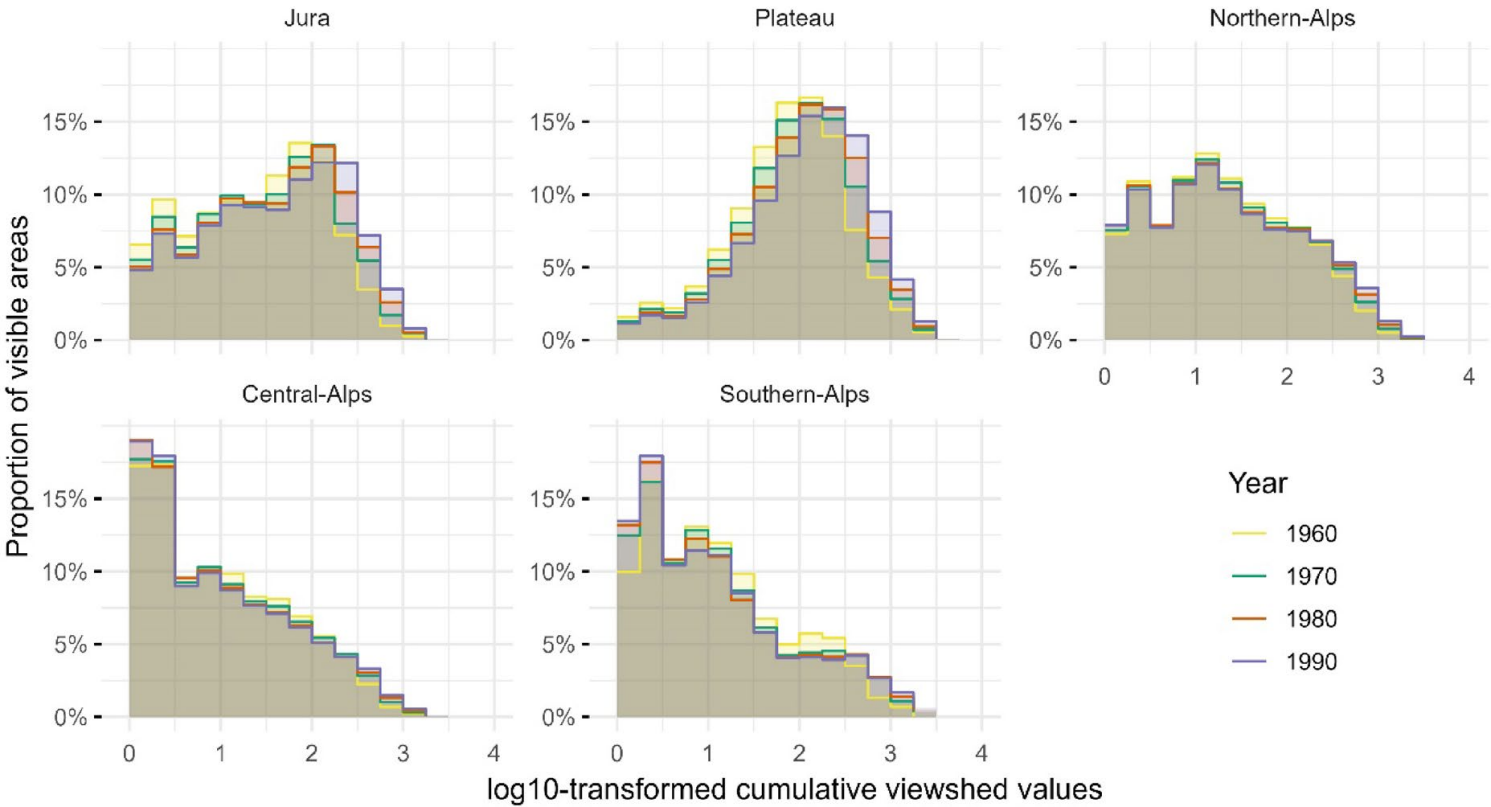
Percentage distribution of visible areas across log10-transformed bins of cumulative building visibility in the biogeographical regions of Switzerland for years 2004, 2014, and 2024.

The distribution of cumulative viewshed values varies strongly across Switzerland’s biogeographical regions, reflecting the combined effects of topography and settlement structure. Over time, regional patterns were sustained, but gradual shifts were observed in visible building numbers across the visible area of the biogeographical regions. The proportional distributions of visible areas across log10-transformed bins of cumulative building visibility illustrate the regional patterns and temporal changes (Figs. 6, 7).

The Plateau region consistently shows the highest visibility values, with the peak of the log10-transformed distribution located in higher bins, [2,2.25) to [2.5,2.75), corresponding to 100–562 visible buildings in approximately 16% of the region’s visible area. This region also shows the highest mean and median cumulative visibility on the original scale, achieving a mean of 363 and a maximum of 4’628 buildings in 2024. In contrast, the Central- and Southern-Alps are characterised by a low cumulative visibility, with distributions peaking in the lowest log10 bins, [0,0.25) or [0.25,0.5), representing one to three visible buildings. These regions also show low mean and median values over the entire study period. For instance, in 2024, the Central-Alps exhibits a mean visibility of only 72 and a median of seven buildings. The Northern-Alps displays a more intermediate profile, with distributions peaking consistently in the [1,1.25) range (i.e., 10–17 visible buildings) across all years. In comparison, the Jura region shows an intermediate distribution, with peak values occurring in mid-range bins, corresponding to 100–316 visible buildings; the mean values are higher than those in the Alpine regions but lower than those in the Plateau.

Distributional shape metrics further highlight regional differences and reveal temporal changes (reported in Supplementary material Table S4). The Plateau exhibits decreasing negatively skewed distributions with increasing positive kurtosis over time, indicating an area with visible building numbers around the upper end of the scale. In contrast, the Alpine regions show increasing positively skewed distributions becoming decreasingly flatter (negative kurtosis), reflecting a high proportion of areas with low cumulative visibility values and a longer tail towards higher values. Notably, the Southern-Alps show increased skewness over time, reaching 0.81 by 2024.

To elucidate where and how distributions evolved over time, the largest absolute changes in cumulative visibility were identified for each region and time period (Supplementary material Table S5). These shifts highlight where the most substantial redistributions in visibility, whether involving increases or decreases. In the Plateau and Jura, the most pronounced changes can be observed in higher log10-transformed value bins, such as [2.5,2.75) and [2.75,3), corresponding to 316–562 and 562–1’000 visible buildings, respectively. For example, the Plateau shows an increase of nearly + 3% in the [2.5,2.75) bin between 1960 and 1970, accompanied by decreases in the lower bins. The Northern-Alps exhibits smaller but still detectable shifts, with changes typically occurring in the log10-transformed range of [1,1.5), representing 10–31 visible buildings. In the Central- and Southern-Alps, the largest increases are mostly confined to the lower log10 bins, [0,0.25) and [0.25,0.5), equivalent to one to three buildings.

Additional results for the years 2004, 2014, and 2024 that incorporate changing forest cover (cf. Supplementary material Figure S7) illustrate how forest expansion further reduces cumulative visibility and provides an indication of the sensitivity of the analysis to vegetation dynamics. Adjusting the maximum visibility distance also affected the cumulative viewshed analysis, resulting in modest shifts in the log10-transformed distributions of visible building counts (cf. Supplementary material Figure S8 to Figure S11). While the overall regional patterns remain intact, increasing the visibility radius notably leads to a smoothing of the distribution shapes for the Alpine regions, where initially left-skewed distributions tend to become more symmetric.

**Fig. 8:**
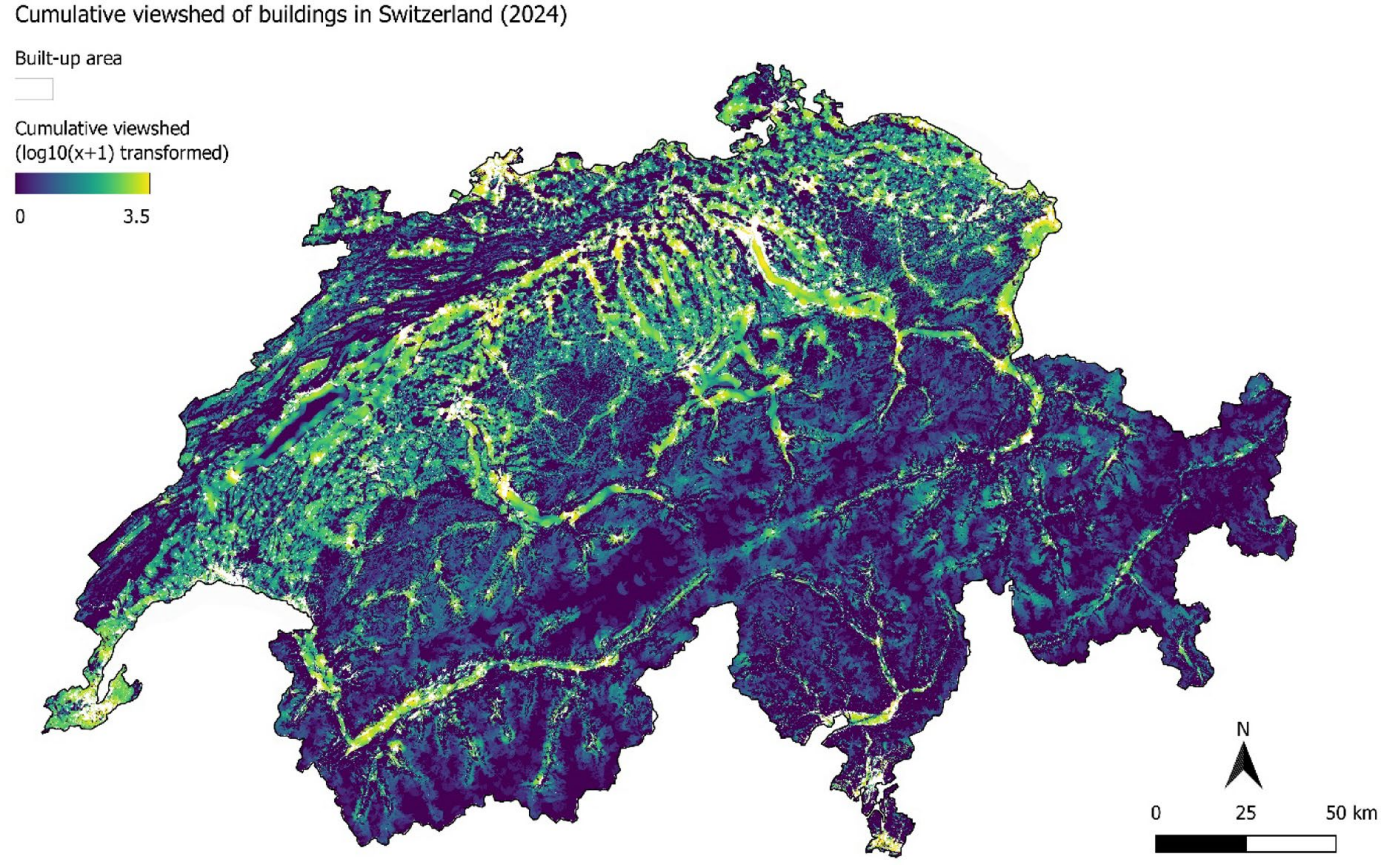
Cumulative building visibility across Switzerland in 2024, involving a log10(x + 1) transformation to compress the range of values while preserving zero values for non-visible areas. Map created using QGIS v3.22.5 (https://qgis.org).

To complement the regional and temporal analyses, Fig. 8 presents a map of the cumulative building visibility in 2024, providing a spatial overview of current viewshed patterns across Switzerland. To improve interpretability and reduce the influence of extreme values, the cumulative visibility values were transformed using a log10(x + 1) scale, maintaining zero values for non-visible areas. The spatial representation reinforces the observed distributional patterns, offering a perspective on the visual connectedness of the current built environment.

## Discussion

The assessment of visual exposure of rural building development in Switzerland over more than six decades reveals distinct spatial patterns and nuanced temporal dynamics. The Plateau region, with its high building density and relatively flat topography, demonstrates a consistently high visual presence of buildings across the landscape. Visual exposure in this region originates from both buildings within and outside of building zones, highlighting the cumulative effect of planned settlements and dispersed rural development. A similar pattern is observed in the Jura region, where, despite a more varied topography, nearly the entire non-forested landscape remains visually connected to at least one building. While the proportion of buildings in building versus non-building zones is similar to that of the Plateau, the Jura’s visibility patterns are less influenced by buildings in building zones, suggesting a greater visual presence of rural buildings. In contrast, the Alpine regions are characterised by substantial areas where no buildings are visible and where visibility is more often determined by rural buildings located outside of building zones.

The results also reveal the temporal evolution of visibility patterns, which, although largely stable in structure, exhibit a gradual increase in visible areas almost entirely due to rural buildings outside of building zones. The most pronounced transitions from non-visible to visible areas occurred during the 1990–2004 period, though this was likely influenced by changes in the underlying building data sources. However, the most substantial visibility expansion attributable to building development occurred between 1960 and 1980, aligning with previous findings on urban sprawl during this period in Switzerland^57^. Another temporal trend involves the reduction in visible landscape areas due to forest expansion. Forest growth emerged as the dominant factor reducing visibility, particularly in the Alps, underscoring the importance of incorporating forest canopy height into viewshed analyses, as advocated by Palmer^5^.

The cumulative viewshed analysis provides additional insights into the intensity and spatial distribution of the visual impacts of buildings across the Swiss landscape. The results confirm a strong contrast between different regions. The Plateau consistently exhibits the highest cumulative visibility, reflecting both the high density and spatial concentration of settlements in this region, as well as the presence of relatively open terrain. In contrast, the Alpine regions, particularly the Central and Southern-Alps, show significantly lower cumulative visibility values, with most areas exposed to only a handful of buildings. These differences are shaped by the interplay between terrain and building dispersion. Over time, the distribution of cumulative visibility has shifted, especially in the Plateau, where increased development has led to growing concentrations of visible buildings in already visually saturated areas. Meanwhile, in the Alpine and Jura regions, changes have been more accentuated for smaller cumulative visibility values; this can be interpreted as a sign of increasing visual influences from rural buildings in non-building zones. These findings underscore the need to consider not only the extent of visibility but also the intensity of visual exposure to evaluate the visual impacts of development with time^31^.

The temporal and regional visibility patterns observed in this study reflect not only settlement trends but also broader policy contexts and demographic dynamics. Following the 1979 Federal Spatial Planning Act, studies have shown that building-zone boundaries effectively concentrated development within designated areas, increasing density inside zones while limiting expansion outside^59^. Yet, development outside of building zones did not disappear altogether but continued at lower rates, often associated with agricultural building needs and rural restructuring^60^. The partial revision of the Spatial Planning Act in 2014 introduced densification as a guiding principle, but research shows that municipal zoning plans still only partially align with higher-level strategic objectives^61^. Demographic shifts further pressured densification. Since 2000, Switzerland has experienced a phase of reurbanisation due to young adults, non-family households and international migrants, while suburbanisation and dispersed development have persisted^62^. These contextual dynamics help to explain the long-term increase in visual exposure, particularly outside building zones, and underline the institutional challenges into effective regulation of urban development.

The viewshed model is based on a series of simplified assumptions and parameter choices that influence the results. One key parameter is the maximum visibility distance. Although a 2 km threshold is consistent with landscape perception and planning literature^56,57^ the actual visibility is affected by contextual factors such as atmospheric conditions, terrain contrast, and human visual limitations. Although adjusting the maximum visibility distance results in different absolute proportions of visible and non-visible areas, the overall temporal trends, regional patterns, and the relative contribution of buildings within and outside of building zones remain consistent. Similarly, the building height was estimated using the footprint area rather than precise data, which is available through alternative datasets not used in this study^63^. Although this approach ensures consistency across historical time steps, it likely underrepresents the visibility of taller buildings in urban or peri-urban settings. Additionally, forest cover was integrated using a static height approximation, assuming a uniform 20 m canopy. While this adjustment improves realistic considerations^52^ it does not account for variations in tree height or vegetation density, nor does it capture seasonal visibility variations or those that occur due to different plant species. A further limitation lies in the treatment of built-up settlement areas. The cumulative visibility was only calculated for open, unbuilt landscapes, and visibility within settlements was excluded from the interpretation. This exclusion avoids overestimating cumulative visibility due to complex obstructions within densely built environments. However, the cumulative visibility along the edges of settlements is most likely overestimated since the first row of buildings can potentially obstruct the line of sight to the interior of the settlement, especially in flat topographies.

Beyond the model assumptions, it is important to recognise conceptual limitations inherent in the use of binary viewshed analysis. As noted in several studies^6,14,27^ binary visibility (i.e., classifying areas simply as visible or not) does not fully capture the complexity of visual experiences. It disregards factors such as object size, contrast, atmospheric clarity, and the viewer’s subjective focus. While incorporating cumulative visibility partially addresses these issues by reflecting the number of visible buildings, it remains purely within the framework of physical visibility. Concepts such as visual magnitude, which accounts for the slope of the terrain, angle of view, and distance^25^ and perception-oriented viewscape models^28^ offer more nuanced approaches to evaluating visual impacts. More recently, detailed representations of visual experience with 3D approaches were developed, for instance to assess dwellers’ window views^64^ or quantify urban openness through visible volumes^65^. These methods achieve high perceptual realism, but they rely on detailed 3D data and intensive computation, currently limiting their application mainly to urban contexts. By contrast, our raster-based approach operates at a higher level of abstraction, sacrificing fine-grained accuracy at specific viewpoints in favour of consistency across six decades and at the scale of entire regions. Together, these approaches illustrate a trade-off between precision and abstraction in visibility modelling and point to promising directions for future research.

Visibility analyses also offer practical guidance for urban design and spatial planning. Metrics of visual exposure help to assess building orientation and integration with landscape elements, indicating when new construction risks becoming visually dominant in sensitive areas. Previous work has demonstrated that visibility-based vegetation screening can inform design interventions that improve the integration of buildings into the surrounding landscape^66^. At a broader scale, distinguishing the visual prominence of buildings inside and outside designated building zones offers evidence for evaluating and refining zoning regulations in regional planning. Identifying areas of high or low cumulative visibility can also assist in preserving open view corridors, allocating public spaces, and designing developments that minimise adverse visual impacts. Likewise, wilderness quality assessments in Switzerland have shown how spatially explicit indicators of human impact and visual intrusion can provide a robust evidence base for landscape protection and monitoring^67^. These perspectives demonstrate how visibility analysis can be integrated into planning frameworks to anticipate long-term visual impacts of settlement development while balancing growth with the preservation of landscape qualities.

## Conclusion

The visual impact assessment of buildings presented in this study adds a novel dimension to the scientific understanding of structural landscape characteristics. By applying binary and cumulative viewshed analyses, the research provides a spatially explicit perspective on how the landscape is visually affected by buildings. This perceptual layer can complement landscape metrics that capture dispersed development, contributing to a comprehensive understanding of urban sprawls from a landscape perspective^57^. As previous research has shown, urban expansion is not only a physical process but also one that is experienced visually, shaping how people perceive and value their surroundings^68^. The relevance of visual openness and scenic integrity has also been underscored in recent efforts to map and protect open spaces in Swiss mountain regions, where stakeholder perceptions play a central role in defining landscape value^69^. Finally, the methods and findings presented here are broadly transferable and can inform spatial planning, landscape monitoring, and conservation efforts in other regions facing similar development pressures. By integrating visibility metrics into planning frameworks, decision-makers can better account for the perceptual dimensions of landscape change and support policies that preserve visual openness and scenic quality in rural environments.

## Supplementary Information

Below is the link to the electronic supplementary material.

Supplementary Material 1

## Data Availability

The datasets generated during and/or analysed during the current study are available from the corresponding author on reasonable request.
