# Supplementary material for: Visual exposure to buildings in Switzerland: spatial patterns and changes over six decades
Source: Sci Rep. 2025 Oct 7;15:34905. doi: 10.1038/s41598-025-18772-7 (PMC12504572; doi:10.1038/s41598-025-18772-7)
Supplement: Supplementary file 1 — Supplementary Material 1 [file 41598_2025_18772_MOESM1_ESM.docx]

# **Supplementary material**

**“Visual exposure to buildings in Switzerland: Spatial patterns and changes over six decades”**

**
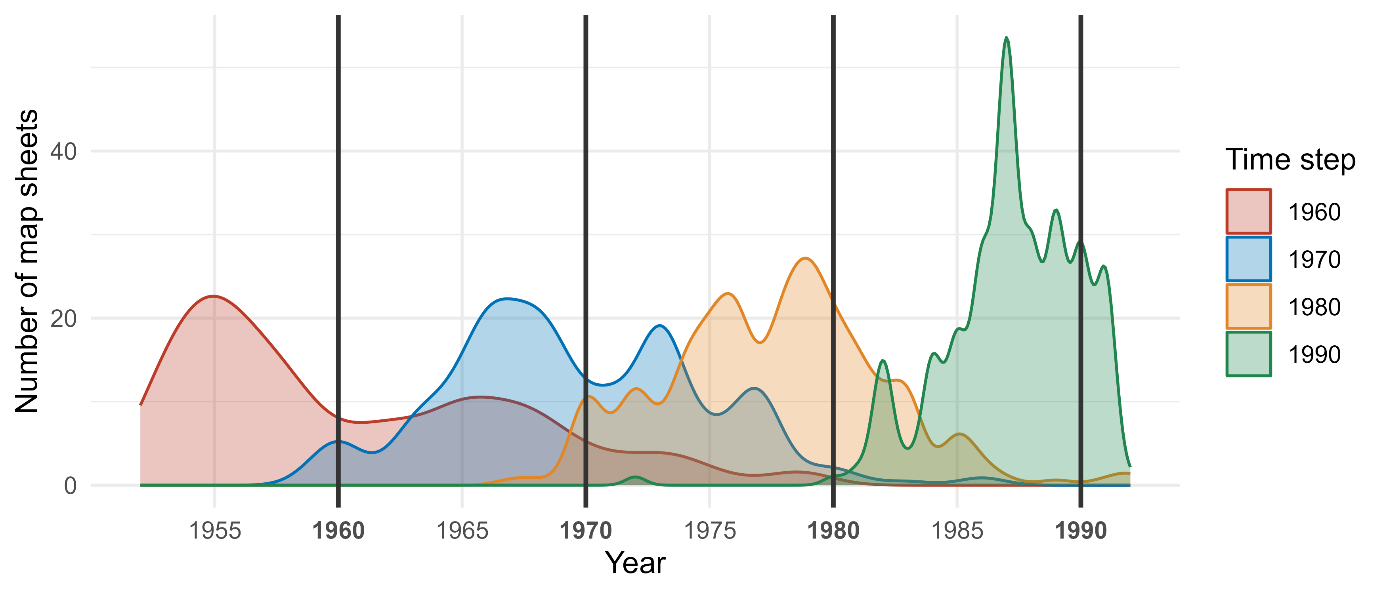
**

Figure S 1: Years of issue of the National Map sheets used for the assembly of a Swiss wide building footprint map at the time steps 1960, 1970, 1980, and 1990.

**
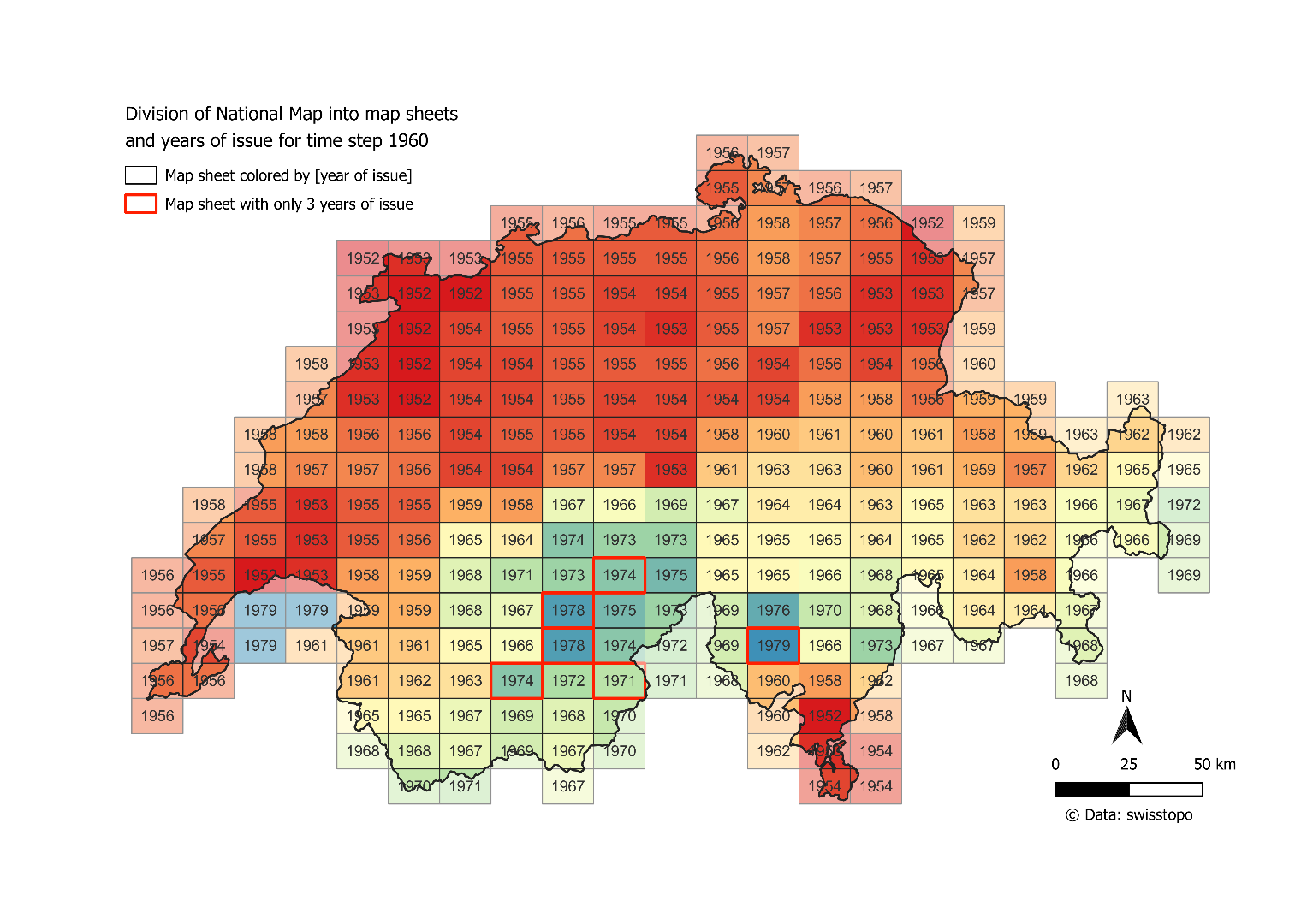
**

Figure S 2: Example of the years of issue of the National Map sheets at the time step of 1960. Map sheets with only 3 years of issue are marked with red outlines. Map created using QGIS v3.22.5 (https://qgis.org).

Table S 1: Building count by year and biogeographical regions of Switzerland. Percentages and growth rates are relative to the yearly Swiss total, the building zones [BZ], and the non-building zones [NBZ].

| Year | Region | Building count [Year] | Percent [Year] | | Percent [Year–NBZ] | Percent [Year–BZ] | Growth [Year] | Growth [Year–NBZ] | Growth [Year–BZ] |
| --- | --- | --- | --- | --- | --- | --- | --- | --- | --- |
| 1960 | Jura | 76'884 | 7.9% | 20.8% | | 79.2% |  |  |  |
|  | Plateau | 461'642 | 47.5% | 19.3% | | 80.7% |  |  |  |
|  | Northern-Alps | 216'923 | 22.3% | 56.6% | | 43.4% |  |  |  |
|  | Central-Alps | 136'159 | 14% | 43.1% | | 56.9% |  |  |  |
|  | Southern-Alps | 79'261 | 8.2% | 44.6% | | 55.4% |  |  |  |
|  | Switzerland | 970'869 | 100% | 33.2% | | 66.8% |  |  |  |
| 1970 | Jura | 101'639 | 8.6% | 20.8% | | 79.2% | 32.2% | 32.2% | 32.2% |
|  | Plateau | 571'558 | 48.6% | 18.8% | | 81.2% | 23.8% | 20.6% | 24.6% |
|  | Northern-Alps | 249'048 | 21.2% | 53.1% | | 46.9% | 14.8% | 7.9% | 23.9% |
|  | Central-Alps | 154'718 | 13.2% | 39.2% | | 60.8% | 13.6% | 3.2% | 21.5% |
|  | Southern-Alps | 98'875 | 8.4% | 37.6% | | 62.4% | 24.8% | 5.2% | 40.5% |
|  | Switzerland | 1'175'838 | 100% | 30.5% | | 69.5% | 21.1% | 11.5% | 25.9% |
| 1980 | Jura | 121'081 | 9% | 21% | | 79% | 19.1% | 20.2% | 18.8% |
|  | Plateau | 677'977 | 50.3% | 18.8% | | 81.2% | 18.6% | 18.4% | 18.7% |
|  | Northern-Alps | 273'291 | 20.3% | 50.6% | | 49.4% | 9.7% | 4.4% | 15.8% |
|  | Central-Alps | 168'180 | 12.5% | 36.1% | | 63.9% | 8.7% | 0.1% | 14.2% |
|  | Southern-Alps | 107'897 | 8% | 34.9% | | 65.1% | 9.1% | 1.2% | 13.9% |
|  | Switzerland | 1'348'426 | 100% | 28.9% | | 71.1% | 14.7% | 8.5% | 17.4% |
| 1990 | Jura | 142'602 | 9.3% | 19.4% | | 80.6% | 17.8% | 8.9% | 20.1% |
|  | Plateau | 791'461 | 51.7% | 17.4% | | 82.6% | 16.7% | 8.2% | 18.7% |
|  | Northern-Alps | 299'624 | 19.6% | 47.9% | | 52.1% | 9.6% | 3.8% | 15.6% |
|  | Central-Alps | 180'632 | 11.8% | 34% | | 66% | 7.4% | 1.2% | 10.9% |
|  | Southern-Alps | 116'521 | 7.6% | 32% | | 68% | 8% | -0.9% | 12.8% |
|  | Switzerland | 1'530'840 | 100% | 26.6% | | 73.4% | 13.5% | 4.7% | 17.1% |
| 2004 | Jura | 169'310 | 9.7% | 19.6% | | 80.4% | 18.7% | 19.4% | 18.6% |
|  | Plateau | 892'121 | 51.3% | 17.6% | | 82.3% | 12.7% | 14.4% | 12.4% |
|  | Northern-Alps | 336'068 | 19.3% | 46.8% | | 53.2% | 12.2% | 9.7% | 14.5% |
|  | Central-Alps | 206'011 | 11.8% | 34.1% | | 65.9% | 14.1% | 14.4% | 13.9% |
|  | Southern-Alps | 135'943 | 7.8% | 33.4% | | 66.6% | 16.7% | 21.6% | 14.3% |
|  | Switzerland | 1'739'453 | 100% | 26.6% | | 73.3% | 13.6% | 13.7% | 13.6% |
| 2014 | Jura | 188'505 | 9.8% | 18.5% | | 81.5% | 11.3% | 5.5% | 12.8% |
|  | Plateau | 1'008'961 | 52.3% | 16.8% | | 83.2% | 13.1% | 7.4% | 14.3% |
|  | Northern-Alps | 368'164 | 19.1% | 43.8% | | 56.2% | 9.6% | 2.5% | 15.8% |
|  | Central-Alps | 221'068 | 11.5% | 31.9% | | 68.1% | 7.3% | 0.4% | 10.9% |
|  | Southern-Alps | 141'751 | 7.3% | 31.6% | | 68.4% | 4.3% | -1.3% | 7% |
|  | Switzerland | 1'928'449 | 100% | 24.9% | | 75.1% | 10.9% | 3.7% | 13.5% |
| 2024 | Jura | 205'901 | 10.2% | 18.9% | | 81.1% | 9.2% | 11.2% | 8.8% |
|  | Plateau | 1'056'847 | 52.4% | 16.7% | | 83.3% | 4.8% | 4.3% | 4.8% |
|  | Northern-Alps | 377'790 | 18.7% | 43.2% | | 56.8% | 2.6% | 1.3% | 3.6% |
|  | Central-Alps | 228'716 | 11.3% | 31.8% | | 68.2% | 3.5% | 2.9% | 3.7% |
|  | Southern-Alps | 148'172 | 7.3% | 31.4% | | 68.6% | 4.5% | 3.7% | 4.9% |
|  | Switzerland | 2'017'426 | 100% | 24.7% | | 75.3% | 4.6% | 3.5% | 5% |

Table S 2: Percentage of biogeographical regions of Switzerland differentiated by visibility to buildings. Visible areas are differentiated as built-up areas (i.e., settlements), areas visible to buildings within building zones only, areas visible to buildings within both building and non-building zones, or areas visible to buildings within non-building zones only. Non-visible areas are differentiated as forested or open non-forested landscapes.

| Region | Total area [km²] | Year | Visible – Built-up area | Visible - Building zone | Visible – Building zone/Non-building zone | Visible – Non-building zone | Non-visible – Forest area | Non-visible |
| --- | --- | --- | --- | --- | --- | --- | --- | --- |
| Jura | 4'171 | 1960 | 1.6% | 1.5% | 32.6% | 16.7% | 43.9% | 3.7% |
|  |  | 1970 | 2.2% | 1% | 33.2% | 16.6% | 43.9% | 3.1% |
|  |  | 1980 | 2.7% | 0.7% | 33.7% | 16.3% | 43.9% | 2.7% |
|  |  | 1990 | 3.3% | 0.6% | 33.5% | 16% | 43.9% | 2.5% |
|  |  | 2004 | 4.3% | 0.5% | 33.2% | 15.9% | 43.9% | 2.2% |
|  |  | 2014 | 4.7% | 0.4% | 32.9% | 15.7% | 44% | 2.2% |
|  |  | 2024 | 5% | 0.5% | 30.3% | 16.5% | 44.2% | 3.4% |
| Plateau | 11'291 | 1960 | 3.8% | 2.7% | 58.7% | 7.3% | 23.2% | 4.3% |
|  |  | 1970 | 5% | 2% | 59% | 6.8% | 23.2% | 4% |
|  |  | 1980 | 6.3% | 1.8% | 58.5% | 6.4% | 23.2% | 3.8% |
|  |  | 1990 | 7.8% | 1.5% | 57.7% | 6.1% | 23.2% | 3.7% |
|  |  | 2004 | 9.6% | 1.4% | 56.3% | 5.9% | 23.2% | 3.6% |
|  |  | 2014 | 10.7% | 1.5% | 55.3% | 5.8% | 23.1% | 3.6% |
|  |  | 2024 | 11.3% | 1.6% | 53.3% | 6.8% | 23.1% | 3.9% |
| Northern-Alps | 10'694 | 1960 | 1.1% | 0.1% | 26.3% | 35.9% | 26.8% | 9.7% |
|  |  | 1970 | 1.4% | 0.1% | 26.6% | 35.9% | 26.8% | 9.3% |
|  |  | 1980 | 1.6% | 0.1% | 26.6% | 35.8% | 26.8% | 9.1% |
|  |  | 1990 | 1.9% | 0.1% | 26.6% | 35.5% | 26.8% | 9.1% |
|  |  | 2004 | 2.5% | 0.1% | 26.3% | 36.2% | 26.8% | 8.2% |
|  |  | 2014 | 2.8% | 0.1% | 26% | 36% | 27.1% | 8% |
|  |  | 2024 | 3% | 0.1% | 22.7% | 36.1% | 29.1% | 9% |
| Central-Alps | 11'300 | 1960 | 0.6% | 0.5% | 18% | 34.4% | 17.7% | 28.8% |
|  |  | 1970 | 0.8% | 0.4% | 18.6% | 34.6% | 17.7% | 28% |
|  |  | 1980 | 0.9% | 0.4% | 18.8% | 35% | 17.7% | 27.2% |
|  |  | 1990 | 1.1% | 0.4% | 18.8% | 34.5% | 17.7% | 27.6% |
|  |  | 2004 | 1.4% | 0.2% | 18.9% | 37% | 17.7% | 24.7% |
|  |  | 2014 | 1.6% | 0.3% | 18.7% | 36.7% | 17.9% | 24.9% |
|  |  | 2024 | 1.7% | 0.3% | 15.6% | 36.6% | 20.1% | 25.7% |
| Southern-Alps | 3'841 | 1960 | 1.1% | 0.4% | 18% | 35% | 33.3% | 12.3% |
|  |  | 1970 | 1.6% | 0.3% | 17.7% | 35.3% | 33.3% | 11.8% |
|  |  | 1980 | 1.9% | 0.2% | 17.5% | 35.1% | 33.3% | 12% |
|  |  | 1990 | 2.2% | 0.2% | 17.2% | 34.9% | 33.3% | 12.1% |
|  |  | 2004 | 2.8% | 0.2% | 16.8% | 36.8% | 33.3% | 10% |
|  |  | 2014 | 3% | 0.2% | 15.5% | 35.6% | 35.4% | 10.3% |
|  |  | 2024 | 3.2% | 0.2% | 12% | 33.2% | 39.6% | 11.8% |

Table S 3: Percentage of biogeographical regions of Switzerland that transitioned between visible to buildings and non-visible to buildings categories over different time periods. Transitions are categorized by areas that became newly visible – differentiated by visibility to buildings in building zones, non-building zones, or both – as well as areas that became non-visible due to forest expansion or other factors.

| Region | Total area [km²] | Time period | Non-visible to visible – Built-up area | Non-visible to visible – Building zone | Non-visible to visible – Building zone/Non-building zone | Non-visible to visible – Non-building zone | Visible to non-visible | Visible to non-visible – Forest area |
| --- | --- | --- | --- | --- | --- | --- | --- | --- |
| Jura | 4'171 | 1960–1970 | 0% | 0.1% | 0.1% | 0.6% | -0.1% | 0% |
|  |  | 1970–1980 | 0% | 0.1% | 0% | 0.5% | -0.1% | 0% |
|  |  | 1980–1990 | 0% | 0% | 0% | 0.2% | -0.2% | 0% |
|  |  | 1990–2004 | 0% | 0% | 0% | 0.4% | -0.1% | 0% |
|  |  | 2004–2014 | 0% | 0% | 0% | 0.2% | -0.3% | -0.6% |
|  |  | 2014–2024 | 0% | 0% | 0% | 0.3% | -1.4% | -1.4% |
| Plateau | 11'291 | 1960–1970 | 0% | 0.1% | 0% | 0.2% | -0.1% | 0% |
|  |  | 1970–1980 | 0% | 0.1% | 0% | 0.2% | -0.1% | 0% |
|  |  | 1980–1990 | 0% | 0% | 0% | 0.1% | -0.1% | 0% |
|  |  | 1990–2004 | 0% | 0% | 0% | 0.1% | 0% | 0% |
|  |  | 2004–2014 | 0% | 0% | 0% | 0.1% | -0.1% | -0.3% |
|  |  | 2014–2024 | 0% | 0% | 0% | 0.1% | -0.5% | -0.6% |
| Northern-Alps | 10'694 | 1960–1970 | 0% | 0% | 0% | 0.8% | -0.4% | 0% |
|  |  | 1970–1980 | 0% | 0% | 0% | 0.6% | -0.4% | 0% |
|  |  | 1980–1990 | 0% | 0% | 0% | 0.5% | -0.4% | 0% |
|  |  | 1990–2004 | 0% | 0% | 0% | 1.2% | -0.3% | 0% |
|  |  | 2004–2014 | 0% | 0% | 0% | 0.4% | -0.3% | -1.3% |
|  |  | 2014–2024 | 0% | 0% | 0% | 0.5% | -1.4% | -3% |
| Central-Alps | 11'300 | 1960–1970 | 0% | 0.1% | 0% | 1.6% | -1% | 0% |
|  |  | 1970–1980 | 0% | 0.1% | 0% | 1.9% | -1.1% | 0% |
|  |  | 1980–1990 | 0% | 0% | 0% | 0.9% | -1.4% | 0% |
|  |  | 1990–2004 | 0% | 0% | 0% | 3.7% | -0.8% | 0% |
|  |  | 2004–2014 | 0% | 0% | 0% | 0.9% | -1.1% | -0.8% |
|  |  | 2014–2024 | 0% | 0% | 0% | 1.1% | -2% | -3% |
| Southern-Alps | 3'841 | 1960–1970 | 0% | 0% | 0% | 1.3% | -0.8% | 0% |
|  |  | 1970–1980 | 0% | 0% | 0% | 1% | -1.2% | 0% |
|  |  | 1980–1990 | 0% | 0% | 0% | 0.8% | -0.9% | 0% |
|  |  | 1990–2004 | 0% | 0% | 0% | 2.7% | -0.7% | 0% |
|  |  | 2004–2014 | 0% | 0% | 0% | 0.5% | -0.8% | -3.2% |
|  |  | 2014–2024 | 0% | 0% | 0% | 0.8% | -2.3% | -5.6% |

Table S 4: Summary of the distribution of log10-transformed cumulative viewshed values by biogeographical regions of Switzerland and year. Reported values include the most frequent bin on the log10-transformed scale (Peak bin [log10]), its non-transformed range (Peak bin [raw]), the proportion of visible area in that bin (Peak bin proportion), the skewness and kurtosis of the log10-transformed distribution, and descriptive statistics of the non-transformed data (Mean [raw], Median [raw], and Max [raw]).

| Region | Year | Peak bin [log10] | Peak bin [raw] | Peak bin proportion | Skewness [log10] | Kurtosis [log10] | Mean [raw] | Median [raw] | Max [raw] |
| --- | --- | --- | --- | --- | --- | --- | --- | --- | --- |
| Jura | 1960 | [1.75,2) | [56–100] | 13.5% | -0.19 | -0.92 | 77 | 30 | 2'417 |
|  | 1970 | [2,2.25) | [100–177] | 13.4% | -0.20 | -0.87 | 97 | 35 | 2'732 |
|  | 1980 | [2,2.25) | [100–177] | 13.3% | -0.25 | -0.86 | 112 | 42 | 2'547 |
|  | 1990 | [2,2.25) | [100–177] | 12.2% | -0.26 | -0.88 | 129 | 47 | 2'621 |
|  | 2004 | [2.25,2.5) | [177–316] | 13% | -0.28 | -0.87 | 147 | 52 | 2'783 |
|  | 2014 | [2.25,2.5) | [177–316] | 12.9% | -0.28 | -0.88 | 161 | 56 | 2'951 |
|  | 2024 | [2.25,2.5) | [177–316] | 12.8% | -0.29 | -0.88 | 176 | 61 | 3'149 |
| Plateau | 1960 | [2,2.25) | [100–177] | 16.7% | -0.51 | 0.26 | 181 | 85 | 4'800 |
|  | 1970 | [2,2.25) | [100–177] | 16.3% | -0.55 | 0.27 | 217 | 103 | 4'645 |
|  | 1980 | [2,2.25) | [100–177] | 16.2% | -0.62 | 0.33 | 250 | 124 | 4'509 |
|  | 1990 | [2.25,2.5) | [177–316] | 16% | -0.68 | 0.37 | 284 | 145 | 4'391 |
|  | 2004 | [2.25,2.5) | [177–316] | 16.1% | -0.73 | 0.44 | 311 | 164 | 4'418 |
|  | 2014 | [2.25,2.5) | [177–316] | 15.8% | -0.75 | 0.45 | 347 | 184 | 4'638 |
|  | 2024 | [2.5,2.75) | [316–562] | 16.1% | -0.78 | 0.48 | 363 | 198 | 4'628 |
| Northern-Alps | 1960 | [1,1.25) | [10–17] | 12.8% | 0.16 | -0.80 | 82 | 18 | 2'740 |
|  | 1970 | [1,1.25) | [10–17] | 12.4% | 0.18 | -0.81 | 93 | 18 | 3'444 |
|  | 1980 | [1,1.25) | [10–17] | 12.1% | 0.20 | -0.84 | 102 | 18 | 3'607 |
|  | 1990 | [1,1.25) | [10–17] | 12% | 0.20 | -0.84 | 112 | 19 | 3'935 |
|  | 2004 | [1,1.25) | [10–17] | 12.2% | 0.22 | -0.84 | 120 | 19 | 4'116 |
|  | 2014 | [1,1.25) | [10–17] | 12.1% | 0.24 | -0.84 | 131 | 19 | 4'308 |
|  | 2024 | [1,1.25) | [10–17] | 12% | 0.26 | -0.84 | 132 | 19 | 4'282 |
| Central-Alps | 1960 | [0.25,0.5) | [1–3] | 17.3% | 0.44 | -0.80 | 49 | 8 | 2'296 |
|  | 1970 | [0,0.25) | [1–1] | 17.7% | 0.49 | -0.75 | 55 | 7 | 2'569 |
|  | 1980 | [0,0.25) | [1–1] | 19% | 0.55 | -0.68 | 58 | 7 | 2'919 |
|  | 1990 | [0,0.25) | [1–1] | 18.9% | 0.56 | -0.68 | 62 | 7 | 3'070 |
|  | 2004 | [0.25,0.5) | [1–3] | 18.6% | 0.61 | -0.56 | 66 | 7 | 3'135 |
|  | 2014 | [0.25,0.5) | [1–3] | 18.2% | 0.63 | -0.51 | 70 | 7 | 3'295 |
|  | 2024 | [0.25,0.5) | [1–3] | 18.5% | 0.66 | -0.45 | 72 | 7 | 3'342 |
| Southern-Alps | 1960 | [0.25,0.5) | [1–3] | 16.2% | 0.51 | -0.56 | 67 | 10 | 1'974 |
|  | 1970 | [0.25,0.5) | [1–3] | 16.2% | 0.63 | -0.45 | 86 | 9 | 2'731 |
|  | 1980 | [0.25,0.5) | [1–3] | 17.5% | 0.69 | -0.39 | 93 | 8 | 2'886 |
|  | 1990 | [0.25,0.5) | [1–3] | 17.9% | 0.71 | -0.35 | 99 | 8 | 3'020 |
|  | 2004 | [0.25,0.5) | [1–3] | 17.1% | 0.73 | -0.23 | 102 | 8 | 3'123 |
|  | 2014 | [0.25,0.5) | [1–3] | 17.2% | 0.76 | -0.19 | 106 | 8 | 3'505 |
|  | 2024 | [0.25,0.5) | [1–3] | 18.8% | 0.81 | -0.12 | 109 | 8 | 3'556 |

Table S 5: Largest year-to-year changes in cumulative viewshed distributions by biogeographical regions of Switzerland. For each time period, the three bins with the highest absolute change in proportion are shown. Each row shows the bin range of cumulative visible buildings in log10- and non-transformed scale, alongside the percentage change in the proportion of the region’s area within that bin.

| Region | Time period | 1st largest bin Δ | 2nd largest bin Δ | 3rd largest bin Δ |
| --- | --- | --- | --- | --- |
| Jura | 1960–1970 | [2.5,2.75) / [316–562]: +2.00% | [1.5,1.75) / [31–56]: -1.28% | [0.25,0.5) / [1–3]: -1.19% |
|  | 1970–1980 | [2.25,2.5) / [177–316]: +2.15% | [2.5,2.75) / [316–562]: +0.92% | [2.75,3) / [562–1000]: +0.87% |
|  | 1980–1990 | [2.25,2.5) / [177–316]: +2.03% | [2,2.25) / [100–177]: -1.10% | [2.75,3) / [562–1000]: +0.92% |
|  | 1990–2004 | [2.75,3) / [562–1000]: +1.00% | [2.25,2.5) / [177–316]: +0.88% | [1.75,2) / [56–100]: -0.79% |
|  | 2004–2014 | [2.5,2.75) / [316–562]: +0.85% | [2.75,3) / [562–1000]: +0.73% | [1.75,2) / [56–100]: -0.58% |
|  | 2014–2024 | [2.75,3) / [562–1000]: +0.93% | [2.5,2.75) / [316–562]: +0.79% | [1.75,2) / [56–100]: -0.50% |
| Plateau | 1960–1970 | [2.5,2.75) / [316–562]: +2.98% | [1.5,1.75) / [31–56]: -1.46% | [1.75,2) / [56–100]: -1.20% |
|  | 1970–1980 | [2.5,2.75) / [316–562]: +1.97% | [2.75,3) / [562–1000]: +1.61% | [1.5,1.75) / [31–56]: -1.30% |
|  | 1980–1990 | [2.75,3) / [562–1000]: +1.79% | [2.5,2.75) / [316–562]: +1.55% | [1.75,2) / [56–100]: -1.26% |
|  | 1990–2004 | [2.75,3) / [562–1000]: +1.61% | [2.5,2.75) / [316–562]: +0.98% | [1.75,2) / [56–100]: -0.84% |
|  | 2004–2014 | [2.75,3) / [562–1000]: +1.47% | [3,3.25) / [1000–1778]: +1.02% | [2,2.25) / [100–177]: -0.90% |
|  | 2014–2024 | [2.75,3) / [562–1000]: +0.83% | [1.75,2) / [56–100]: -0.62% | [2.5,2.75) / [316–562]: +0.57% |
| Northern-Alps | 1960–1970 | [2.75,3) / [562–1000]: +0.60% | [2.5,2.75) / [316–562]: +0.49% | [1,1.25) / [10–17]: -0.41% |
|  | 1970–1980 | [2.75,3) / [562–1000]: +0.52% | [1.25,1.5) / [17–31]: -0.45% | [0,0.25) / [1–1]: +0.36% |
|  | 1980–1990 | [2.75,3) / [562–1000]: +0.45% | [0.25,0.5) / [1–3]: -0.29% | [3,3.25) / [1000–1778]: +0.27% |
|  | 1990–2004 | [0,0.25) / [1–1]: -0.61% | [2.75,3) / [562–1000]: +0.37% | [2.5,2.75) / [316–562]: +0.30% |
|  | 2004–2014 | [2.75,3) / [562–1000]: +0.39% | [3,3.25) / [1000–1778]: +0.36% | [2,2.25) / [100–177]: -0.30% |
|  | 2014–2024 | [0.25,0.5) / [1–3]: +0.44% | [0.5,0.75) / [3–5]: +0.29% | [0,0.25) / [1–1]: -0.23% |
| Central-Alps | 1960–1970 | [1,1.25) / [10–17]: -0.73% | [2.5,2.75) / [316–562]: +0.59% | [1.5,1.75) / [31–56]: -0.50% |
|  | 1970–1980 | [0,0.25) / [1–1]: +1.33% | [1.5,1.75) / [31–56]: -0.44% | [0.25,0.5) / [1–3]: -0.38% |
|  | 1980–1990 | [0.25,0.5) / [1–3]: +0.75% | [0.5,0.75) / [3–5]: -0.57% | [2.5,2.75) / [316–562]: +0.25% |
|  | 1990–2004 | [0,0.25) / [1–1]: -1.05% | [1,1.25) / [10–17]: +0.68% | [0.25,0.5) / [1–3]: +0.63% |
|  | 2004–2014 | [0.25,0.5) / [1–3]: -0.38% | [0.5,0.75) / [3–5]: +0.24% | [0,0.25) / [1–1]: +0.23% |
|  | 2014–2024 | [0.25,0.5) / [1–3]: +0.30% | [1.5,1.75) / [31–56]: -0.20% | [1.25,1.5) / [17–31]: +0.19% |
| Southern-Alps | 1960–1970 | [0,0.25) / [1–1]: +2.50% | [2.75,3) / [562–1000]: +1.38% | [2,2.25) / [100–177]: -1.31% |
|  | 1970–1980 | [0.25,0.5) / [1–3]: +1.34% | [0,0.25) / [1–1]: +0.71% | [1.25,1.5) / [17–31]: -0.66% |
|  | 1980–1990 | [0.75,1) / [5–10]: -0.79% | [1.25,1.5) / [17–31]: +0.47% | [0.25,0.5) / [1–3]: +0.44% |
|  | 1990–2004 | [0.75,1) / [5–10]: +1.33% | [0,0.25) / [1–1]: -1.32% | [0.25,0.5) / [1–3]: -0.82% |
|  | 2004–2014 | [0,0.25) / [1–1]: +0.97% | [1,1.25) / [10–17]: -0.31% | [2.5,2.75) / [316–562]: -0.29% |
|  | 2014–2024 | [0.25,0.5) / [1–3]: +1.55% | [1.25,1.5) / [17–31]: -0.64% | [0,0.25) / [1–1]: -0.51% |


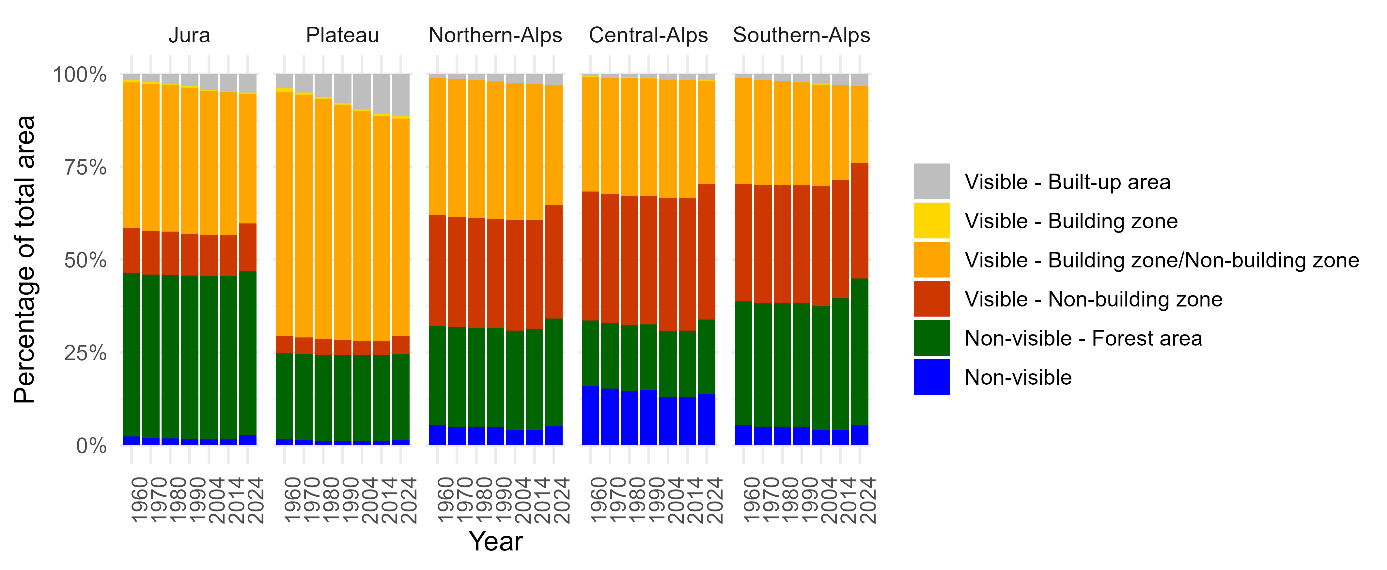


Figure S 3: Percentage of biogeographical regions of Switzerland visible or non-visible to buildings using 5 km maximum visibility distance.


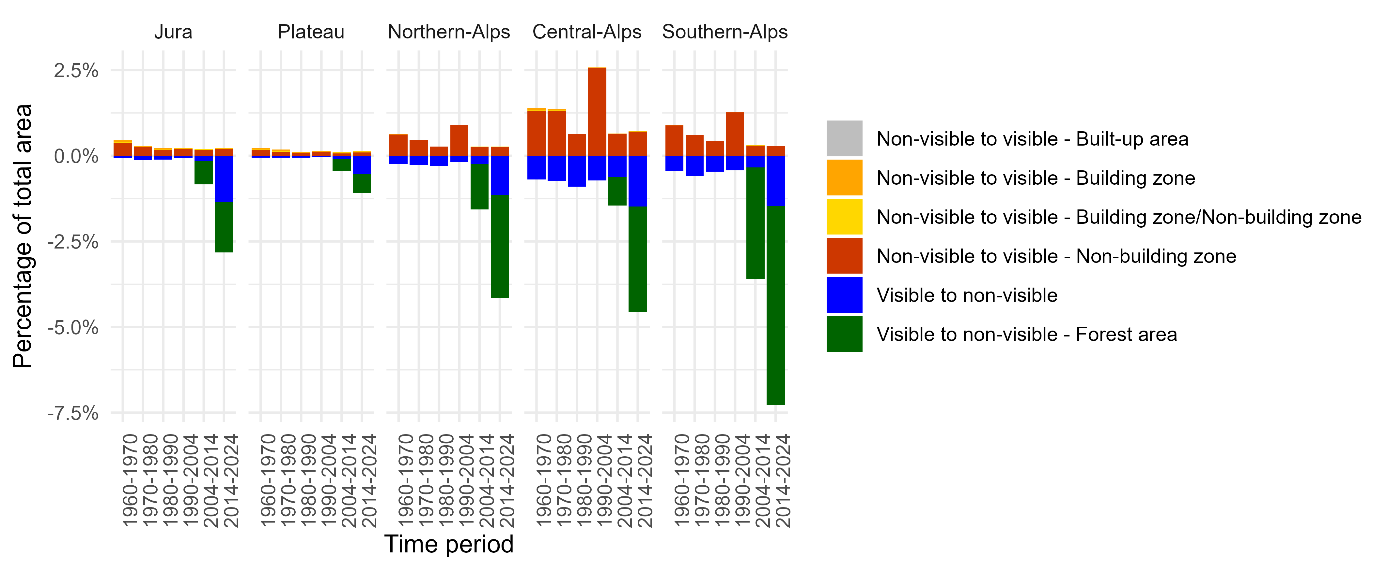


Figure S 4: Transitions between visible and non-visible categories over different time periods using 5 km maximum visibility distance.


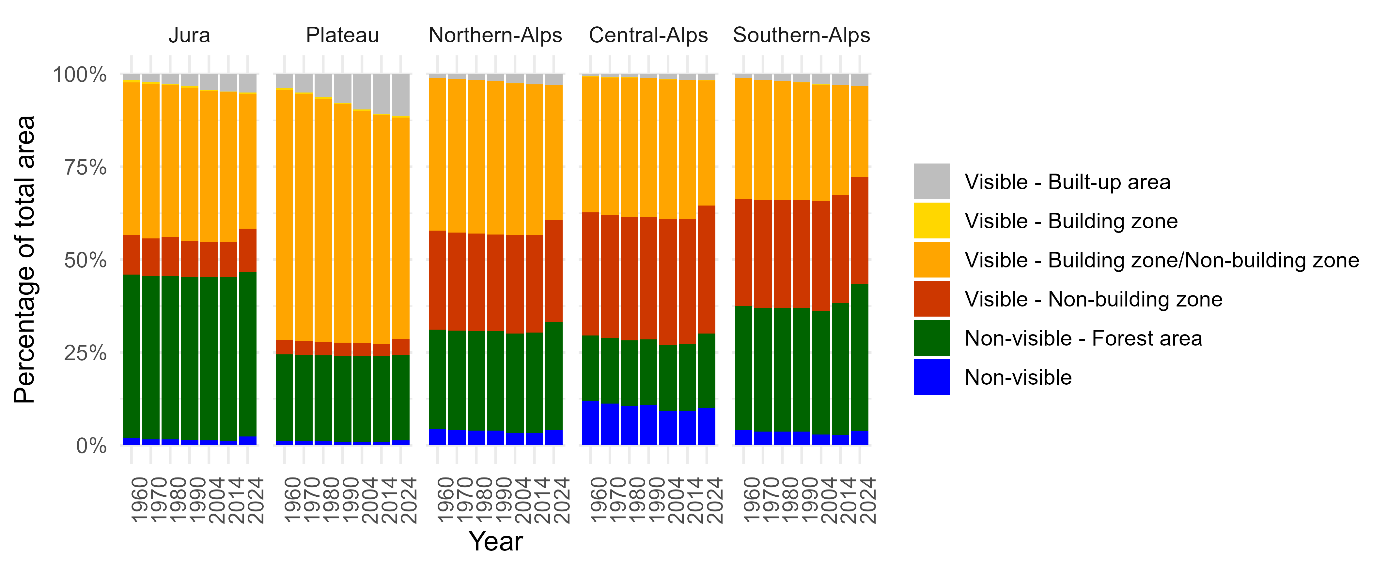


Figure S 5: Percentage of biogeographical regions of Switzerland visible or non-visible to buildings using 8 km maximum visibility distance.


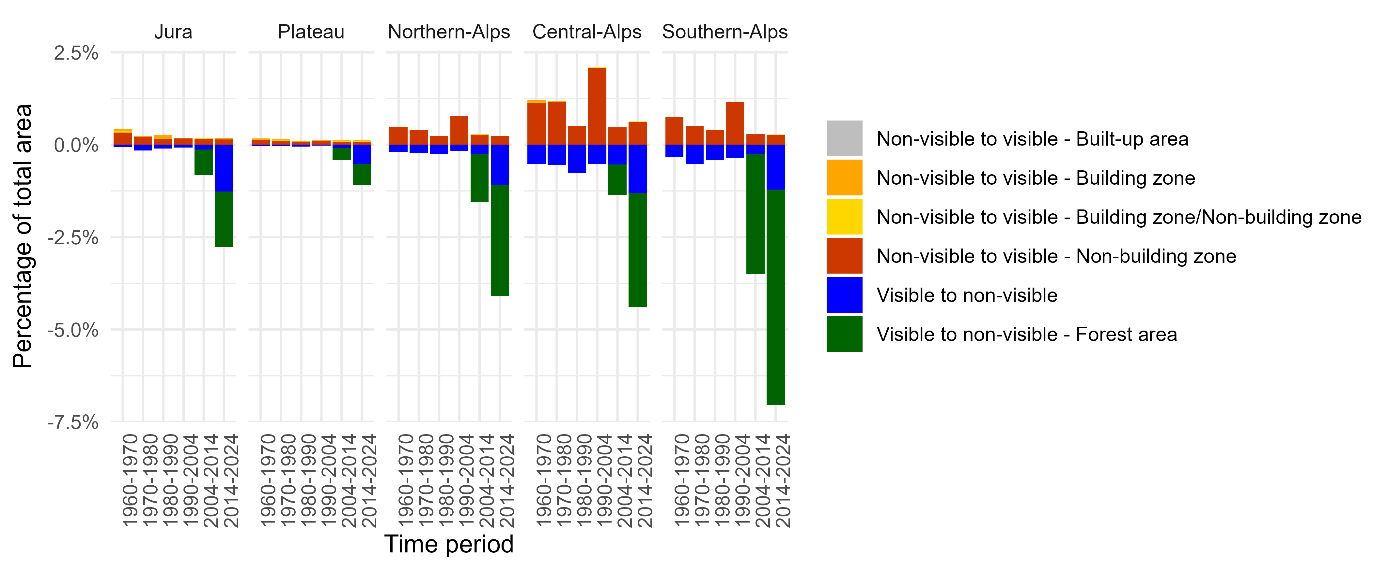


Figure S 6: Transitions between visible and non-visible categories over different time periods using 8 km maximum visibility distance.


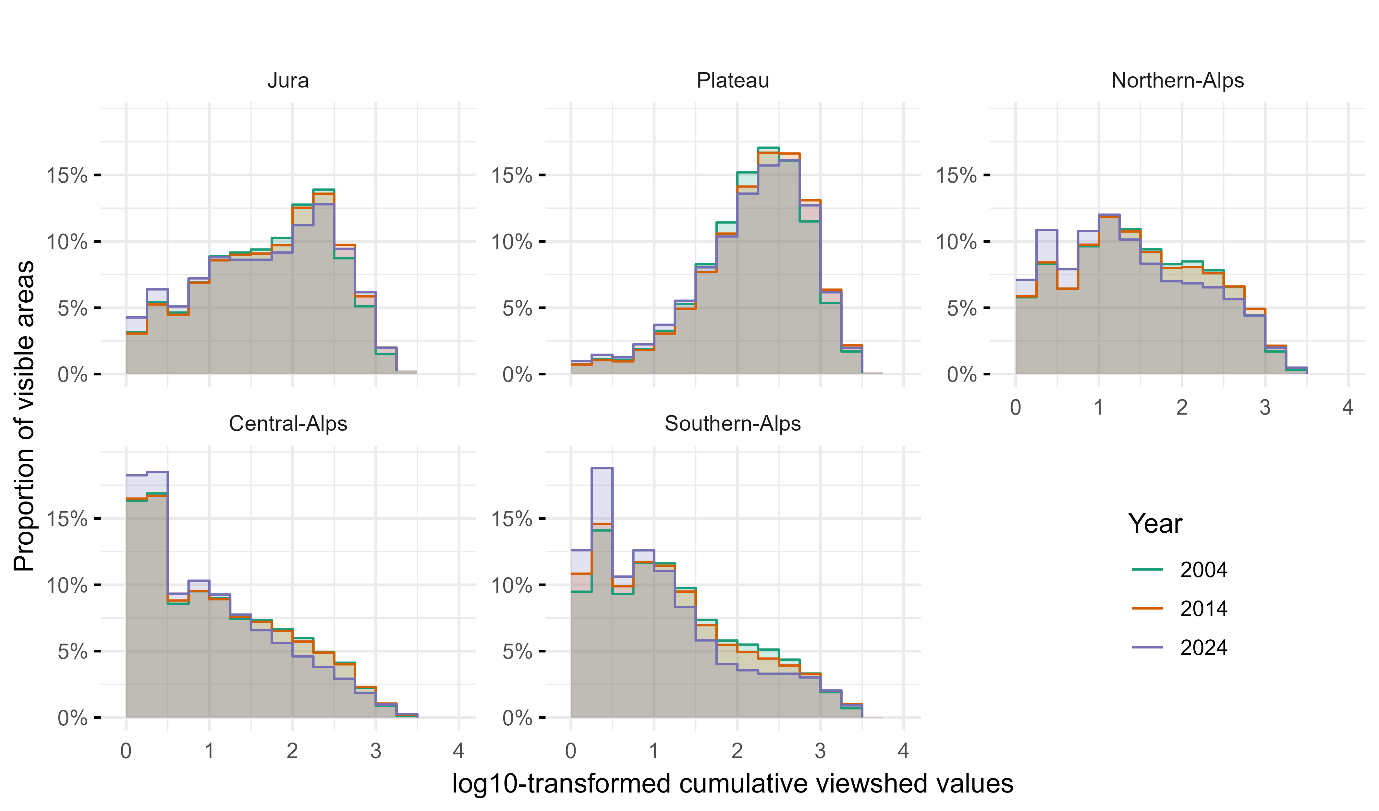


Figure S 7: Percentage distribution of visible areas across log10-transformed bins of cumulative building visibility in the biogeographical regions of Switzerland for the years 2004, 2014, and 2024 using 2 km maximum visibility distance and incorporating changing forest cover.


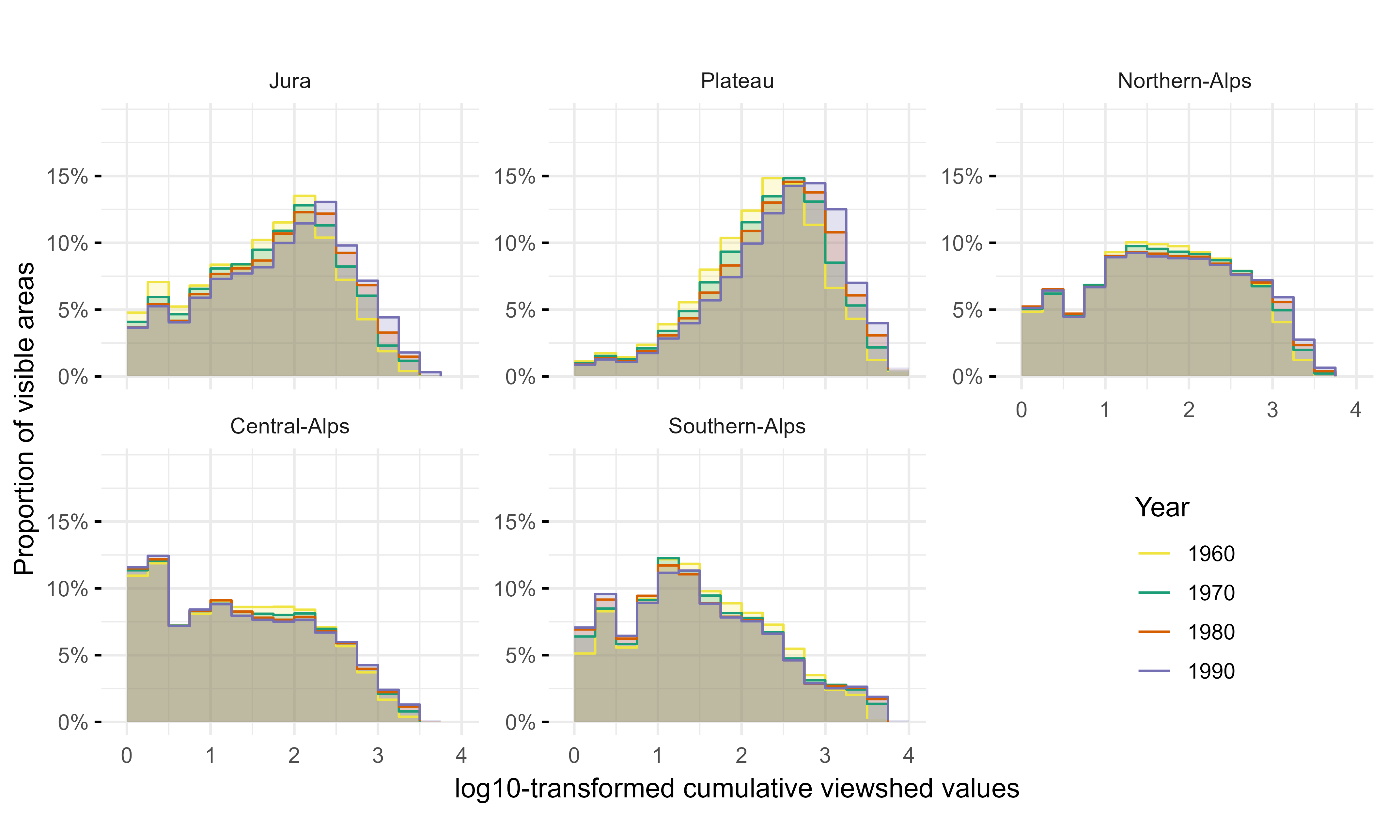


Figure S 8: Percentage distribution of visible areas across log10-transformed bins of cumulative building visibility in the biogeographical regions of Switzerland for the years 1960, 1970, 1980, and 1990 using 5 km maximum visibility distance.


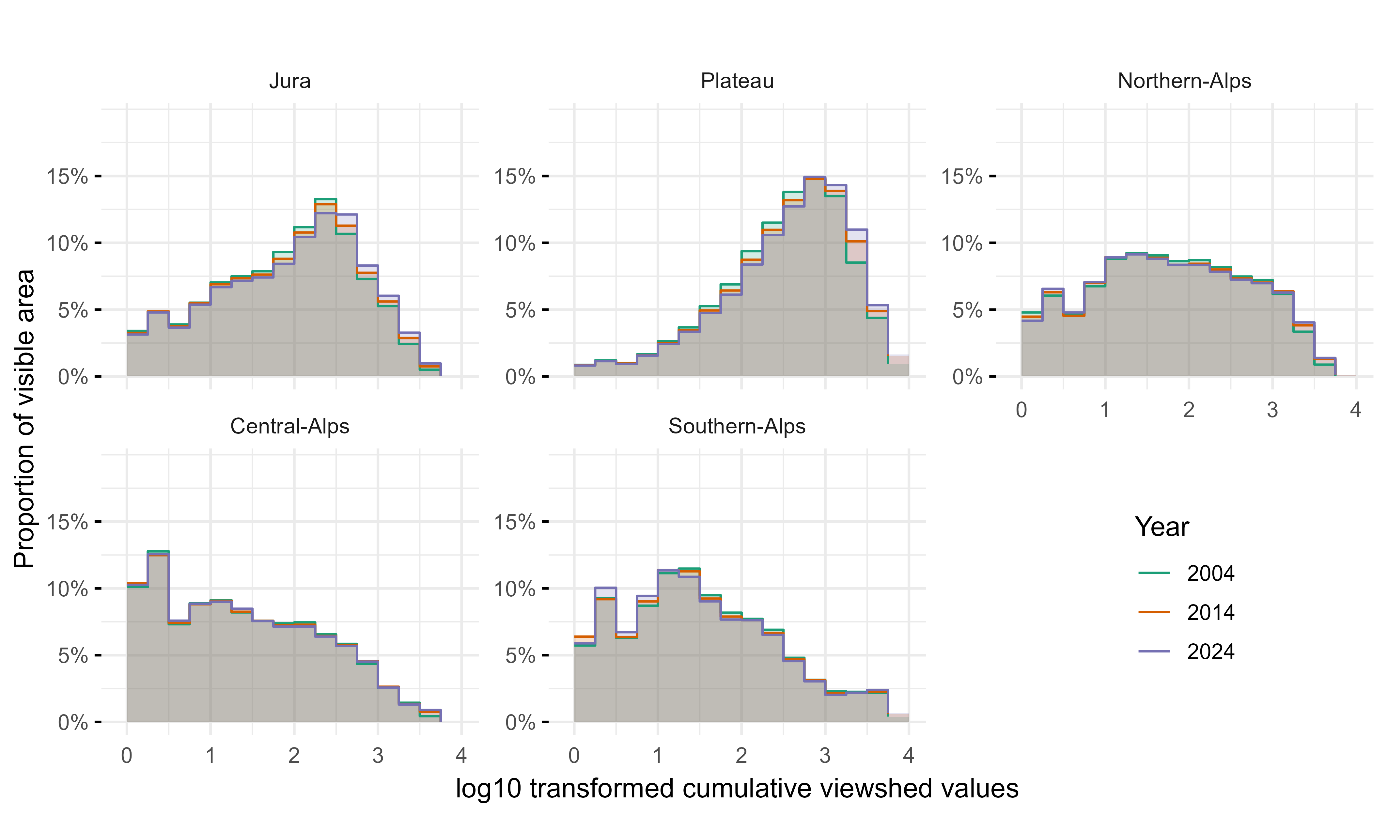


Figure S 9: Percentage distribution of visible areas across log10-transformed bins of cumulative building visibility in the biogeographical regions of Switzerland for the years 2004, 2014, and 2024 using 5 km maximum visibility distance.


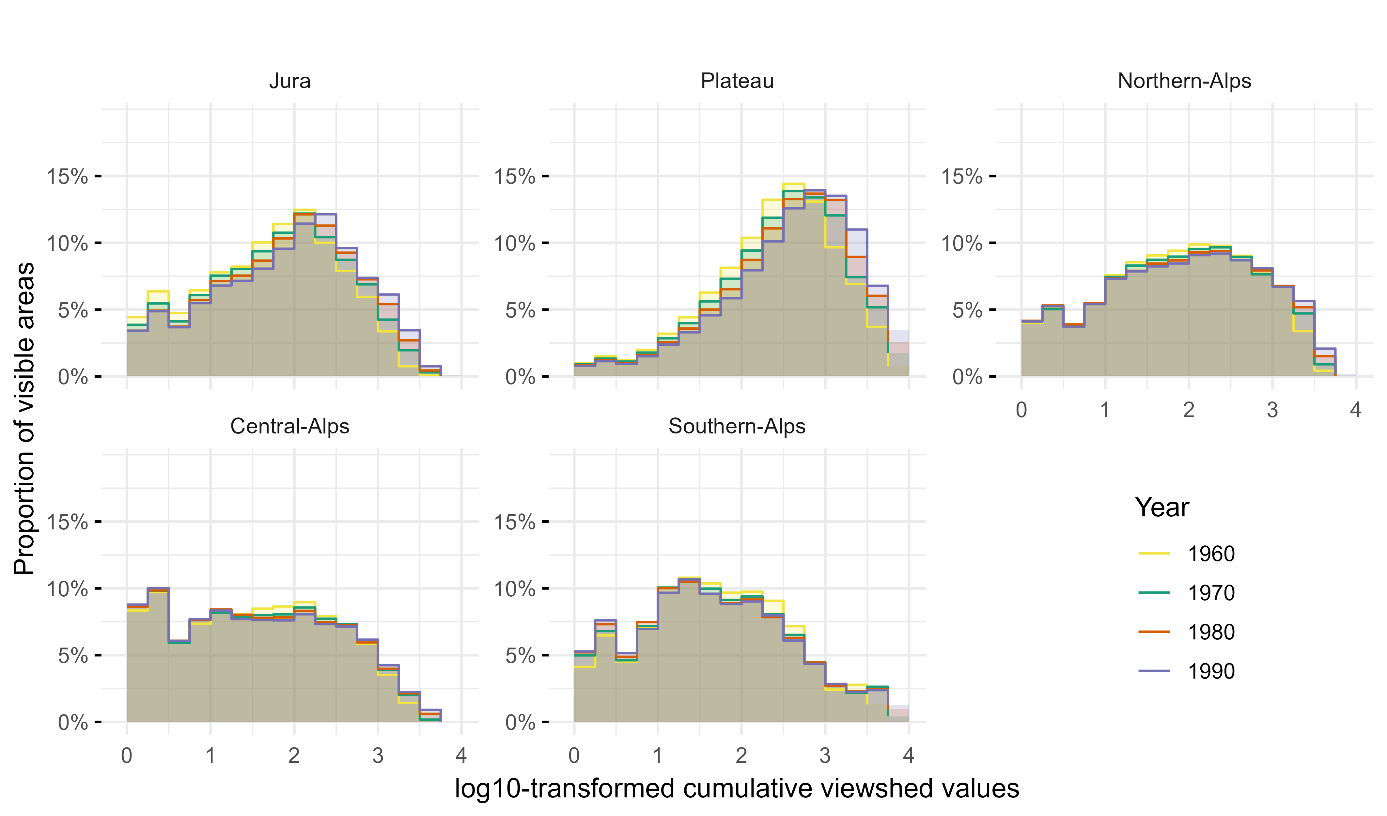


Figure S 10: Percentage distribution of visible areas across log10-transformed bins of cumulative building visibility in the biogeographical regions of Switzerland for the years 1960, 1970, 1980, and 1990 using 8 km maximum visibility distance.


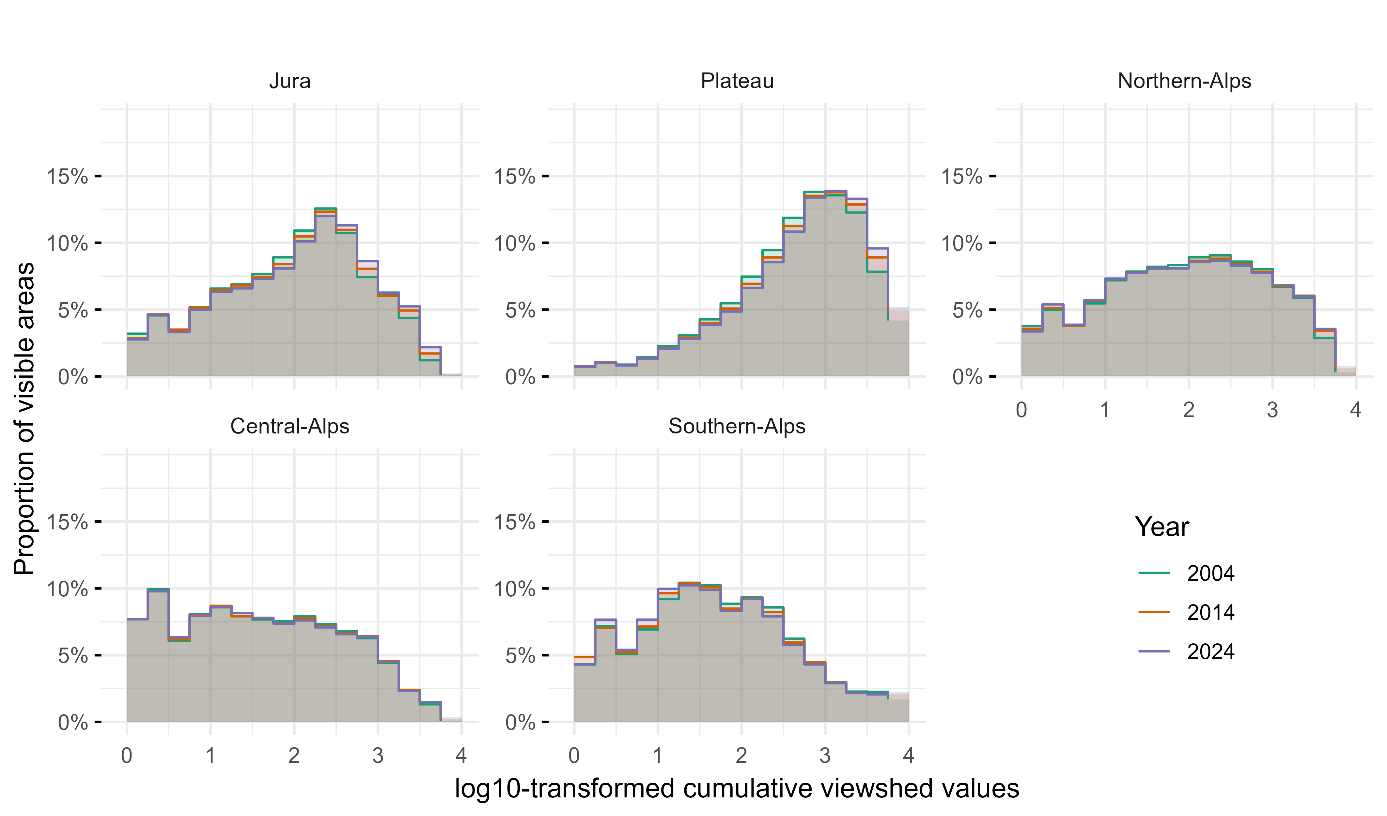


Figure S 11: Percentage distribution of visible areas across log10-transformed bins of cumulative building visibility in the biogeographical regions of Switzerland for the years 2004, 2014, and 2024 using 8 km maximum visibility distance.
